# Purely organic electroluminescent material realizing 100% conversion from electricity to light

**DOI:** 10.1038/ncomms9476

**Published:** 2015-10-19

**Authors:** Hironori Kaji, Hajime Suzuki, Tatsuya Fukushima, Katsuyuki Shizu, Katsuaki Suzuki, Shosei Kubo, Takeshi Komino, Hajime Oiwa, Furitsu Suzuki, Atsushi Wakamiya, Yasujiro Murata, Chihaya Adachi

**Affiliations:** 1 Institute for Chemical Research, Kyoto University, Uji, Kyoto 611-0011, Japan; 2 Center for Organic Photonics and Electronics Research, Kyushu University, 744 Motooka, Nishi, Fukuoka 819-0395, Japan; 3 JST, ERATO, Adachi Molecular Exciton Engineering Project; 4 International Institute for Carbon Neutral Energy Research (WPI-I2CNER), Kyushu University, 744 Motooka, Nishi, Fukuoka 819-0395, Japan

## Abstract

Efficient organic light-emitting diodes have been developed using emitters containing rare metals, such as platinum and iridium complexes. However, there is an urgent need to develop emitters composed of more abundant materials. Here we show a thermally activated delayed fluorescence material for organic light-emitting diodes, which realizes both approximately 100% photoluminescence quantum yield and approximately 100% up-conversion of the triplet to singlet excited state. The material contains electron-donating diphenylaminocarbazole and electron-accepting triphenyltriazine moieties. The typical trade-off between effective emission and triplet-to-singlet up-conversion is overcome by fine-tuning the highest occupied molecular orbital and lowest unoccupied molecular orbital distributions. The nearly zero singlet–triplet energy gap, smaller than the thermal energy at room temperature, results in an organic light-emitting diode with external quantum efficiency of 29.6%. An external quantum efficiency of 41.5% is obtained when using an out-coupling sheet. The external quantum efficiency is 30.7% even at a high luminance of 3,000 cd m^−2^.

## Abstract (web summary)

Organic light-emitting diodes promise a more environment-friendly future for light sources, but many use rare metals. Here, the authors present an approach that achieves external quantum efficiency over 40% by realising 100% up-conversion from triplet to singlet excitons and thus 100% radiative emission.

## Graphical abstract

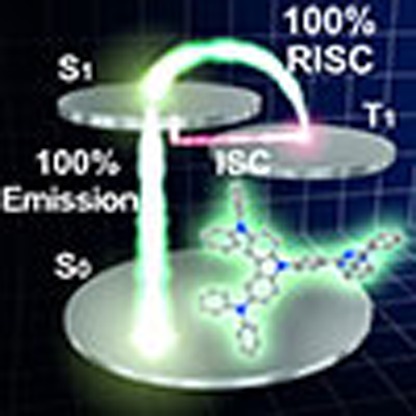

Electroluminescence (EL) was initially studied for fundamental interest^1^,^2^,^3^. Tang and VanSlyke^4^ subsequently applied EL in organic light-emitting diodes (OLEDs), obtaining an external EL quantum efficiency (EQE) of 1% for a double-layer fluorescent OLED. OLEDs have since been studied extensively, with particular focus on increasing their efficiency in display and lighting applications. EQE (*η*_EQE_) is the product of the internal quantum efficiency (IQE) and light out-coupling factor (*η*_out_), *η*_EQE_=IQE × *η*_out_. *η*_out_ is typically 20–30% (refs 5, 6, 7, 8). IQE is obtained from IQE=*β* × *γ* × *Φ*_PL_, where *β* is the exciton generation factor resulting in photons, *γ* is the carrier balance ratio of holes and electrons, and *Φ*_PL_ is the photoluminescence (PL) quantum yield (PLQY). Singlet and triplet excitons are theoretically generated in a 1:3 ratio by electronic excitation^3^. Triplet excitons typically dissipate as heat rather than being converted into photons. Because only singlet excitons generate photons, the resulting *β* for fluorescent OLEDs is limited to 25%. Even under conditions where *γ* and *Φ*_PL_ are 100%, the theoretical maximum *η*_EQE_ of the resulting OLED is only 5–7.5%.

Increasing *β* and *η*_out_ are therefore important challenges to maximize EQE. *β* can be increased directly using triplet excitons. The heavy-atom effect can convert triplet excitons into phosphorescence. Intersystem crossing (ISC) involves transition from the singlet to triplet state, and can theoretically result in 100% of excitons being extracted as photons. The EQE of the first device based on such a concept in 1998 was 4% (ref. 9), and subsequently reached 19% in 2001 (ref. 10). EQEs exceeding 20% are now frequently reported for optimized devices incorporating well-designed materials^11^. However, phosphorescent materials for OLEDs are currently restricted to Pt and Ir complexes, which have limited availability and are expensive.

Triplet excitons can also potentially be exploited by reverse ISC (RISC), which involves transition from the lowest energy triplet state (T_1_) to the lowest excited singlet state (S_1_)^12^,^13^,^14^,^15^,^16^. Converting triplet excitons to singlet excitons allows all excitons to be used to generate photons. This strategy has typically not been effective, because S_1_ is at a much higher energy than T_1_. Spin inversion is also required for this transition. However, fabrications of efficient OLEDs based on RISC have been attempted in recent years^17^,^18^,^19^,^20^,^21^. Unlike normal fluorescence, the fluorescence resulting from triplet–singlet transition is delayed, so we term it thermally activated delayed fluorescence (TADF).

TADF occurs by two successive processes, shown in Fig. 1a as green arrows; RISC from T_1_ to S_1_, and subsequent radiative decay from S_1_ to the ground state (S_0_). RISC is effective when the energy difference between S_1_ and T_1_ (Δ*E*_ST_) is small. The theoretical background (Supplementary Note 1) reveals that small Δ*E*_ST_ is achieved by separating highest occupied molecular orbital (HOMO) and lowest unoccupied molecular orbital (LUMO) distributions. However, this HOMO–LUMO separation decreases the oscillator strength (*f*) between S_1_ and S_0_, which lowers PLQY^21^.

Here we report an organic TADF material, 9-[4-(4,6-diphenyl-1,3,5-triazin-2-yl)phenyl]-*N*,*N*,*N*′,*N*′-tetraphenyl-9*H*-carbazole-3,6-diamine, named DACT-II (Fig. 1b). DACT-II consists of electron-donating diphenylaminocarbazole and electron-accepting triphenyltriazine moieties, chemically bonded at a torsion angle *α* (Fig. 1b). We will demonstrate that both nearly zero Δ*E*_ST_, which is smaller than the thermal energy at room temperature, and large *f*, resulting in a PLQY of 100%, are simultaneously achieved by precisely controlling the HOMO and LUMO distributions (Fig. 1c and Supplementary Fig. 1, the geometries were optimized at the PBE0/6-31G(d) level^22^,^23^ using the Gaussian 09 software package^24^), although the two factors are considered to be in a trade-off relationship. The small Δ*E*_ST_ and large *f* of DACT-II allow ‘all injected holes and electrons to be used in fluorescence', resulting in an IQE of 100%. **DACT-II-based** **OLEDs** exhibit a maximum EQE of 29.6% in the absence of out-coupling treatment, indicating an IQE of ∼100%. The only loss of quantum efficiency in the device is via light out-coupling. A maximum EQE of 41.5% is obtained simply using an out-coupling sheet. Importantly, DACT-II also allows the typical substantial decrease in EQE with increasing luminance (efficiency roll-off) to be overcome. In DACT-II, S_1_ and T_1_ excitons are rapidly converted to photons because of its large *f* and small Δ*E*_ST_. The rapid removal of both S_1_ and T_1_ excitons in the optimized DACT-II-based OLEDs suppresses triplet–triplet annihilation (TTA) and singlet–triplet annihilation, which are an origin of roll-off. Very high EQE up to 30.7% is achieved at 3,000 cd m^−2^.

## Results

### Organic light-emitting diodes

**Figure 1:**
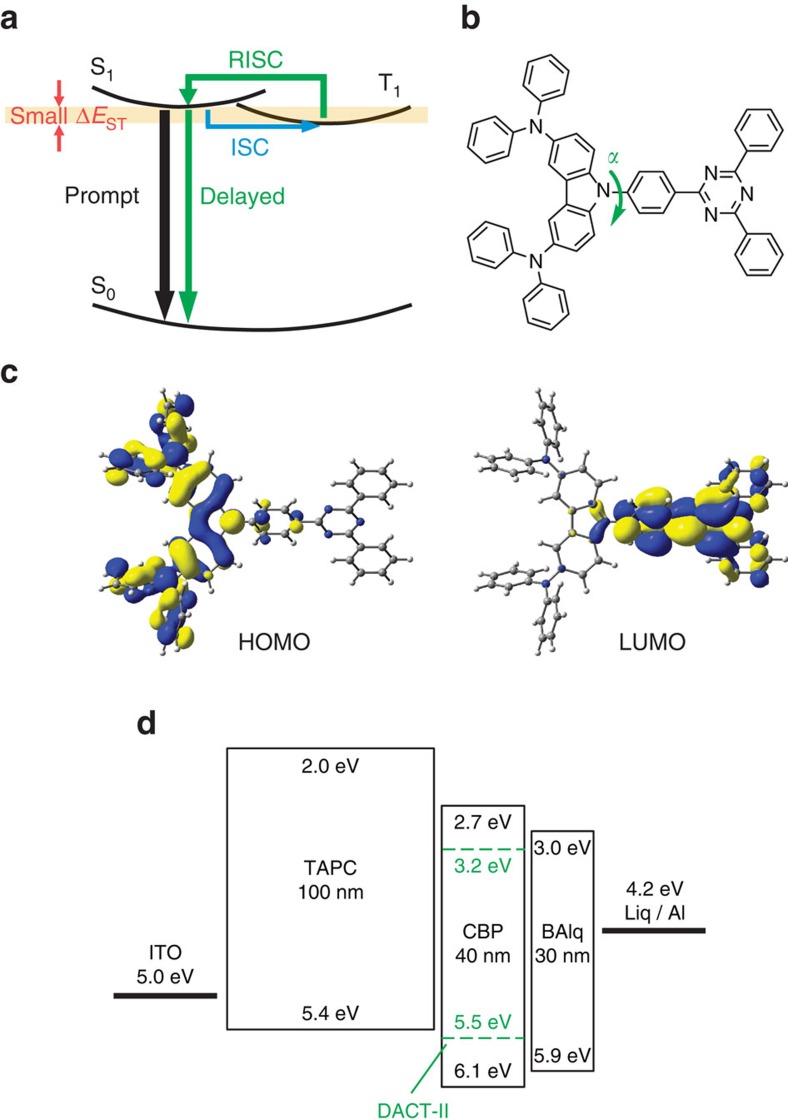
DACT-II. (**a**) Schematic illustration of the TADF process. (**b**) Structure of DACT-II. (**c**) HOMO and LUMO distributions in DACT-II calculated at the PBE0/6-31G(d) level of theory. (**d**) Device structure and energy band diagram of **DACT-II-*x* OLEDs**. For the **DACT-II-100 OLED**, the EML consisted of DACT-II without CBP. Energy band diagrams of the reference fluorescent and phosphorescent OLEDs and other **DACT-II-*x* OLEDs** are shown in Supplementary Fig. 4.

DACT-II, which consists only of C, H and N, was synthesized as described in Supplementary Note 2 and Supplementary Fig. 2. DACT-II has favourable thermal properties, with melting and decomposition temperatures of 266 °C and 484 °C, respectively. The sublimation temperature under vacuum was 308 °C. The glass transition temperature (*T*_g_) of bulk amorphous DACT-II (∼6 mg) measured by conventional differential scanning calorimetry (DSC) at heating rates of 1–100 K min^−1^ was 151–159 °C. We also attempted DSC measurements for DACT-II in vacuum-deposited amorphous thin films with a thickness of 100 nm (∼20 ng). The ultrasensitive measurements were successful using very fast heating rates of 500–3,000 K s^−1^, giving *T*_g_ of 192–197 °C. *T*_g_ of the thin films was higher than that of bulk samples, although it would be decreased by extrapolating to a lower heating rate. The higher *T*_g_ probably reflects the higher thermal stability of vacuum-deposited thin films than bulk samples^25^ (Supplementary Note 3). DACT-II was then used as an emitting material for OLEDs (**DACT-II devices**). Supplementary Fig. 3 shows the materials for the devices; *N*,*N*′-di(naphthalen-1-yl)-*N*,*N*′-diphenyl-[1,1′-biphenyl]-4,4′-diamine (NPD) or 4,4′-(cyclohexane-1,1-diyl)bis(*N*,*N*-di-*p*-tolylaniline) (TAPC) was used for the hole-transport layer (HTL), ([1,1′-biphenyl]-4-yloxy)bis((2-methylquinolin-8-yl)oxy)aluminium (BAlq) or 3,3′′,5,5′′-tetra(pyridin-3-yl)-1,1′:3′,1′′-terphenyl (BmPyPhB) for the electron-transport layer (ETL), lithium quinolin-8-olate (Liq) for the electron-injection layer (EIL) and 4,4′-di(9*H*-carbazol-9-yl)-1,1′-biphenyl (CBP) for the host material of the emitter layer (EML). The device structure was indium tin oxide (ITO)/HTL (100 nm)/EML (40 nm)/ETL (30 nm)/Liq/Al (Fig. 1d and Supplementary Fig. 4). CBP doped with *x* wt% DACT-II was used for the EML. The devices are termed **DACT-II-*****x*** **devices**, where *x* corresponds to the respective wt% of DACT-II. Hereafter, the **DACT-II-*****x*** **devices**, containing TAPC and BAlq as HTL and ETL, respectively, are discussed unless otherwise stated. The energy-level diagram of the devices is shown in Fig. 1d. A neat DACT-II layer was also used for the EML in the **DACT-II-100 device**. The results and band diagrams for devices containing NPD or BmPyPhB are included in Table 1, Supplementary Tables 1 and 2, and Supplementary Figs 4 and 5. Typical fluorescent and phosphorescent devices were also fabricated for reference using *mer* tris(8-hydroxyquinoline) aluminum(III) (Alq_3_) and *fac* tris(2-phenylpyridine) iridium(III) (Ir(ppy)_3_) as emitters, respectively. The structures of the **Alq**_3_ **device** and **Ir(ppy)**_3_ **device** are shown in Supplementary Fig. 4.

**Figure 2:**
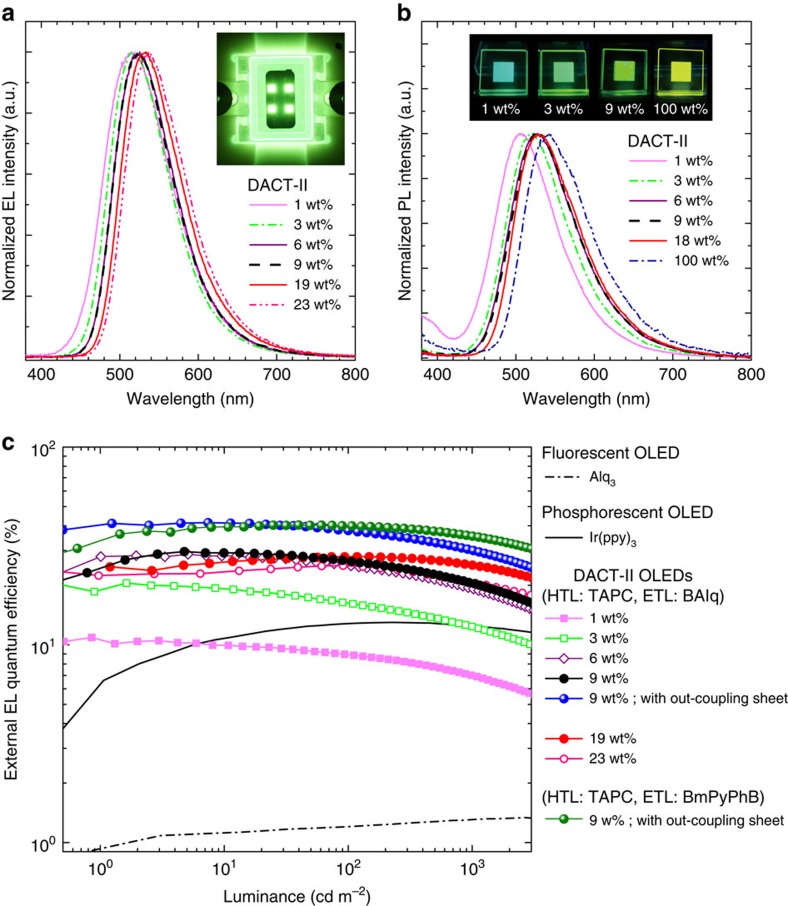
EL and PL of DACT-II. (**a**) EL spectra for the **DACT-II-*x* devices**. A photograph of EL emission for the **DACT-II-9 device** is also shown. (**b**) PL spectra of DACT-II in CBP films and a neat DACT-II film. Photographs of PL emission are also shown. (**c**) EQE versus luminance for the **DACT-II-*x* devices**. EQE values of the **Alq_3_ device** and **Ir(ppy)_3_ device** are also shown.

Figure 2a shows EL spectra of **DACT-II devices** at a current density of 1 mA cm^−2^. The OLEDs, containing DACT-II concentrations of 1 to 23 wt%, all emit green EL. The emission maxima (*λ*_max_) range from 516 to 537 nm, with a small red shift observed with increasing DACT-II concentration. *λ*_max_ of PL spectra has a stronger concentration dependence, as shown in Fig. 2b (see Supplementary Note 4). EL spectra with a logarithmic EL intensity scale are shown in Supplementary Fig. 6. The **DACT-II-1** **device** contains emission from TAPC and/or CBP (Supplementary Figure 6 also shows the PL spectra of the respective materials). However, no obvious radiative emission from any other layer is detected for any of the other devices. This indicates that excitons are confined within the emission layer, despite the absence of blocking layers. In addition, no emission originating from CBP is observed, except for the **DACT-II-1 device**. This indicates that excitons are well-confined on DACT-II molecules, by direct charge injection and/or complete energy transfer from the CBP host. In Fig. 2c, EQE is shown as a function of luminance. The EQE for representative **DACT-II-*****x*** **devices** are given in Table 1 (Data for all OLEDs without and with out-coupling sheets are listed in Supplementary Tables 1 and 2, respectively). The maximum EQE of 29.6% is obtained at 5 cd m^−2^ (5 μA cm^−2^) for the **DACT-II-9 device** (filled black circles in Fig. 2c). EQEs of a typical fluorescent **Alq**_**3**_ **device** and phosphorescent **Ir(ppy)**_**3**_ **device** are also shown in Fig. 2c, as black dot-dashed and solid lines, respectively. Maximum EQEs of the **Alq**_**3**_ and **Ir(ppy)**_**3**_ **devices** are 1.3% and 13.0%, respectively. Maximum EQEs of all fabricated **DACT-II devices** exceed those of the **Alq**_**3**_ and **Ir(ppy)**_**3**_ **devices**, except for the **DACT-II-1 device**. EQE values of the **DACT-II-9 device** are extremely large, even at the luminance levels of practical applications; 22.8% at 500 cd m^−2^ (for display applications) and 16.2% at 3,000 cd m^−2^ (for lighting applications) (Table 1). Although EQE decreases with increasing luminance, the efficiency roll-off is overcome by increasing the concentration of DACT-II in the EML. Figure 2c and Table 1 show that EQE values of 26.9% and 21.8% are obtained at 500 cd m^−2^ and 3,000 cd m^−2^, respectively, for the **DACT-II-19 device**.

Although the devices are relatively thick (Fig. 1d), the **DACT-II devices** exhibit low power consumption, especially at high DACT-II concentrations. The turn-on voltage (voltage at 1 cd m^−2^) decreased with increasing DACT-II concentration and was 2.6 V for the **DACT-II-19 device**. This suggests that with increasing DACT-II concentration, charges tend to be injected directly into DACT-II molecules rather than via the CBP host, as supposed from Fig. 1d.

### Photoluminescence quantum yield

We investigate the origin of the excellent EQE of DACT-II by first discussing the PLQY (Supplementary Note 5). The PLQY of DACT-II in toluene is 46.0%, which decreases to 26.6% on bubbling O_2_ through the solution. Bubbling with Ar excludes O_2_ and results in a PLQY of 63.7%. This suggests that some excitons, transferred to T_1_ via ISC, are quenched by dissolved O_2_ (refs 17, 18). In the Ar-purged system, excitons in T_1_ are not quenched and revert to S_1_ through RISC, resulting in a higher PLQY. Phosphorescence can be discounted as demonstrated below. The PLQY of the neat film is only 43.6±4%, but PLQY values of 101.1±4% are obtained for the 9 wt% DACT-II-doped CBP thin films, when DACT-II is directly excited. The PLQYs are 100.3±4% for the 9 wt% doped films even when CBP is excited, indicating that all the excitons transfer from CBP to DACT-II without any loss (ultraviolet–visible spectra of DACT-II and CBP films are given in Supplementary Fig. 7).

### Exciton generation factor

**Figure 3:**
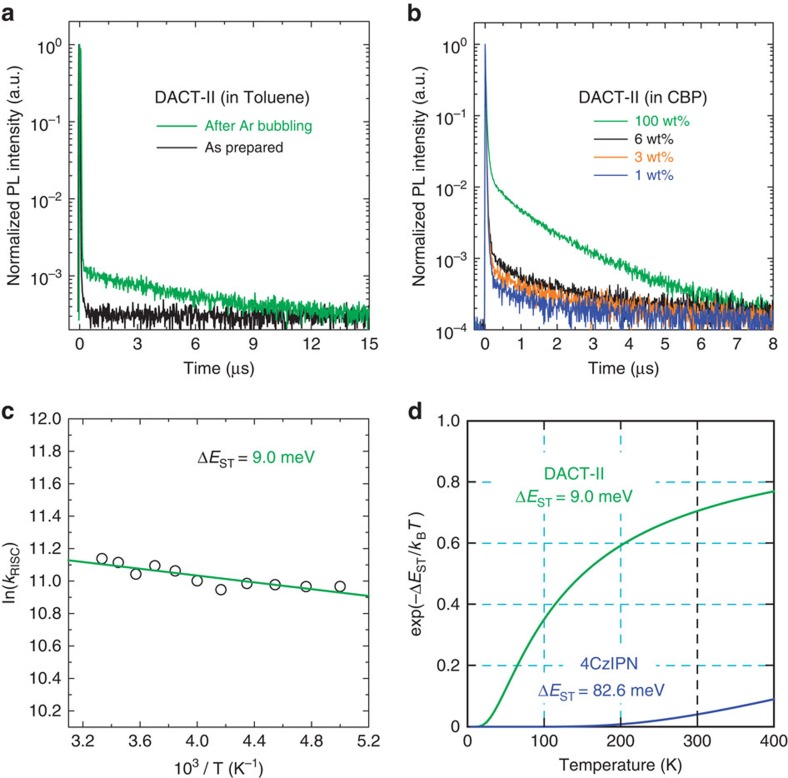
TADF characteristics of DACT-II. (**a**) Transient PL decay characteristics of DACT-II in toluene at room temperature. (**b**) Doping concentration dependence of transient PL decay of DACT-II doped into CBP thin films at room temperature. (**c**) Arrhenius plot of the RISC rate constant (*k*_RISC_). Δ*E*_ST_ of 9.0 meV was obtained from least-squares fitting (solid line). (**d**) Temperature dependence of Boltzmann factor 
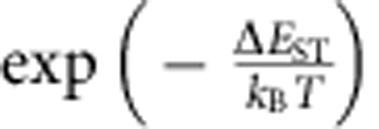
for DACT-II (Δ*E*_ST_=9.0 meV) and 4CzIPN (Δ*E*_ST_=82.6 meV).

Another important factor influencing EQE is *β*, that is, the singlet/triplet branching ratio. In PL experiments, excitation produces excitons in S_1_. Figure 3a shows the transient PL decay of DACT-II in toluene. When O_2_ is removed by Ar bubbling, two components with prompt and delayed PL decays are observed. This differs from the result for the as-prepared solution containing O_2_, in which only the prompt decay component is observed. The results indicate that the prompt decay arises from fluorescence directly from the S_1_-to-S_0_ transition. The delayed component arises from phosphorescence from T_1_ via ISC, or delayed fluorescence from S_1_ through ISC and subsequent RISC, because it is quenched by the O_2_ triplet state in the presence of O_2_ (refs 17, 18). Continuous transitions between S_1_ and T_1_ are theoretically possible, but also result in either phosphorescence or delayed fluorescence.

Prompt and delayed PL decay components are also observed for thin films. Figure 3b shows the doping concentration dependence of the transient PL decays for DACT-II-doped CBP thin films (The temperature dependence is shown in Supplementary Fig. 8). Both prompt and delayed components are observed for all films, and the delayed component increases with increasing DACT-II concentration. Phosphorescence and delayed fluorescence are easily distinguished from the temperature dependence of the transient PL decay. Delayed fluorescence exhibits an increase in delayed emission with increasing temperature. Conversely, inverse temperature dependence is found in the case of phosphorescence. The intensity of the delayed component is absent at 10 K but gradually increases with increasing temperature (Supplementary Fig. 8), indicating that the delayed emission is thermally activated. Thus, the delayed component originates from TADF.

TTA is another possible origin of delayed fluorescence, which can also improve OLED efficiency^26^,^27^,^28^,^29^. However, *β* obtained by TTA is theoretically limited to 62.5%. The IQE, and therefore *β*, in this study reached 100% as clearly shown below, confirming that the delayed fluorescence originates from RISC.

The radiative rate constants for the prompt (*k*_p_) and delayed (*k*_d_) components, rate constants for ISC (*k*_ISC_) and RISC (*k*_RISC_), and total (*Φ*_PL_) and fraction of delayed (*Φ*_PL_^d^) emission quantum yields are shown in Table 2 (see Supplementary Note 6 for the analysis). Δ*E*_ST_ determined from the Arrhenius plot of *k*_RISC_ is 9.0 meV (Fig. 3c). The currently reported TADF emitting material with the highest efficiency is the carbazolyldicyanobenzene derivative 4CzIPN^19^, whose experimental Δ*E*_ST_ is 82.6 meV. Δ*E*_ST_ of DACT-II is markedly smaller, reflecting the temperature dependence of *k*_RISC_.

Δ*E*_ST_ of DACT-II is smaller than the thermal energy at 300 K (25.9 meV). Figure 3d compares the temperature dependence of the Boltzmann factors, exp(–Δ*E*_ST_/*k*_B_*T*), for DACT-II and 4CzIPN. The Boltzmann factor of DACT-II at 300 K is 0.71, much larger than that of 4CzIPN, 0.041. This indicates that RISC occurs more readily in DACT-II than 4CzIPN. The temperature dependence of the Boltzmann factors suggests effective up-conversion from T_1_ to S_1_ for DACT-II even at low temperature. This is supported by the total PLQY remaining at ∼100% at lower temperatures, as shown in Table 2. T_1_ excitons are more easily up-converted to S_1_ at higher temperature. This is an advantage of DACT-II TADF systems over other TADF and phosphorescent systems, the latter two systems cannot exhibit high PLQY across a wide temperature range.

The PL spectra of the prompt and delayed PL decay components are shown in Supplementary Fig. 9. These spectra are indistinguishable within experimental error, irrespective of temperature. This supports the fact that the delayed component originates from TADF, not phosphorescence. In phosphorescent OLEDs, the energy corresponding to Δ*E*_ST_ is lost during ISC. Although this does not affect the quantum yield, such energy loss is avoided (or there is even an energy gain) during RISC in TADF systems by exploiting the thermal energy at room temperature. This results in lower power consumption.

Time-dependent density functional theory^30^,^31^ (TD-DFT) calculations indicate that the torsion angle (*α*) between the donor and acceptor moieties affects the spatial overlap of the HOMO and LUMO (Supplementary Fig. 1, *α*=0° for planar configuration and 90° for perpendicular configuration). The value of *f* decreases with increasing *α*, so precise control of *f* by adjusting *α* is important in designing highly luminescent TADF compounds. The value of *f* for DACT-II (0.2400) is higher than that for 4CzIPN (0.0708). S_0_, T_1_ and S_1_ have energy minima at *α* of 48°, 85° and 89°, respectively. Subtracting the energy minimum of T_1_ (at *α*=85°) from that of S_1_ (at *α*=89°) gives a calculated Δ*E*_ST_ of 5.2 meV (using TD-DFT with the PBE0/6-31G(d) basis set^22^,^23^). This is smaller than the Δ*E*_ST_ of 11.3 meV calculated for 4CzIPN at the same level of theory. We realized both ∼100% up-conversion from T_1_ to S_1_ and ∼100% radiative emission, that is to say, 100% conversion from electricity to light, through the fine-tuning of Δ*E*_ST_ and *f*, by precisely controlling the overlap of the HOMO and LUMO distributions in DACT-II. In real OLEDs, the energy levels of S_1_ and T_1_ likely have distributions, and Δ*E*_ST_ may fluctuate. This is due to the distributions of conformations and environments of individual DACT-II molecules because they are in an amorphous state.

### Orientation of transition dipole moment

**Figure 4:**
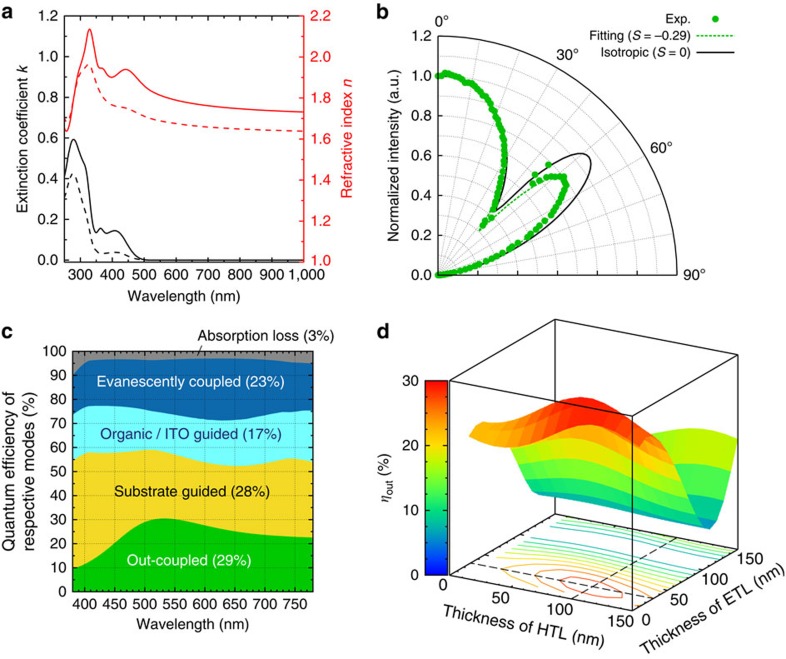
Orientation of transition dipole moment and out-coupling. (**a**) Results of VASE measurements; extinction coefficient *k* (black) and refractive index *n* (red). The solid and dashed lines represent spectra for the ordinary and extraordinary optical constants, respectively. (**b**) Results of angular-dependent PL experiments. (**c**) Optical simulations for the **DACT-II-9 device**. The numbers in parentheses for respective modes are obtained by the integration with respect to emission wavelengths with weighting of the PL intensity. (**d**) Dependence of the out-coupling mode on the thicknesses of hole and electron-transport layers.

The orientation of transition dipole moments of emitter molecules greatly affects out-coupling efficiency^32^,^33^,^34^,^35^. Here the orientation of DACT-II was quantified by two methods, variable angle spectroscopic ellipsometry (VASE)^36^,^37^,^38^ and angular-dependent PL measurements^32^,^33^,^39^,^40^ (Supplementary Note 7). Figure 4a shows the results of VASE measurements. The ordinary and extraordinary optical constants differ in the spectra, indicating that the orientation of DACT-II molecules is not random. Quantitative analysis indicates that DACT-II tends to be parallel to the substrate with an order parameter *S* of −0.32±0.01. The result is confirmed by the angular-dependent PL measurements (Fig. 4b), which gave *S*=−0.29±0.05, agreeing well with the above VASE result.

### Out-coupling and carrier balance ratio

We performed electrical and optical simulations^41^,^42^,^43^ for the **DACT-II-9** **device** (Supplementary Note 8). Electrical simulations revealed that the injected electrons and holes effectively recombine in the EML near the interface with the HTL. Figure 4c shows the results of optical simulations for the **DACT-II-9** **device**. IQE=100% and *S*=−0.29 were used for the simulation. The contributions of respective optical modes are given as a function of emission wavelengths. EQE can be calculated by integrating the fraction of out-coupled mode with respect to emission wavelengths with weighting of the PL intensity in Fig. 2b. Figure 4d shows the dependence of EQE on HTL and ETL thicknesses (see Supplementary Fig. 10 for all modes). The maximum EQE of 29.0±0.9% was obtained for HTL and ETL thicknesses of 100 nm and 30 nm, respectively (for *S*=−0.29±0.05). The simulation results agree well with the experimental maximum EQE of 29.6%. The agreement clearly confirms the IQE≈100%; that is, *β*≈*γ*≈*Φ*_PL_≈100% for the conditions of maximum EQE.

Thus far, we have made no effort to improve the out-coupling efficiency. The current OLEDs have a high *η*_out_=*η*_EQE_/(*γ* × *β* × *Φ*_PL_)=29.6/(1.0 × 1.0 × 1.0)=29.6%. The IQE is already ∼100%, so an increase in out-coupling efficiency is required to further increase EQE. Several methods have been reported to achieve this^44^,^45^,^46^,^47^,^48^. We opted for a simple, economical attachment of an out-coupling sheet to the OLED. Using an out-coupling sheet (Supplementary Fig. 11 and Supplementary Note 9) with a microlens array that also contained light-scattering particles yielded EQE values of 41.5% at maximum (filled blue circles in Fig. 2c), and 37.6% and 30.7% at 500 cd m^−2^ and 3,000 cd m^−2^, respectively (filled green circles in Fig. 2c, see also Table 1 and Supplementary Table 2).

## Discussion

We obtained a TADF material, DACT-II. OLEDs containing DACT-II have ∼100% IQE, that is, 100% conversion from electricity to light. DACT-II has excellent thermal properties, and devices containing DACT-II are expected to perform well across a wide temperature range. By further optimizing device structures and surrounding materials, as seen for phosphorescent^11^,^49^ and TADF-based^40^ OLEDs, **DACT-II devices** are expected to deliver much higher performance, such as higher EQE at high luminance and lower power consumption. Improving the out-coupling efficiency will further increase the EQE of **DACT-II-based OLEDs**.

## Methods

### Summary of DACT-II synthesis

DACT-II was synthesized according to Supplementary Fig. 2 and Supplementary Note 2 with a 26% total yield. ^1^H and ^13^C nuclear magnetic resonance (NMR) spectra were recorded using JEOL ECA 600 MHz (Japan) and Bruker Avance III 800 MHz (Germany) spectrometers. DACT-II was purified by silica gel column chromatography using dichloromethane/hexane as an eluent, recrystallization and temperature-gradient sublimation.

### Device fabrication and characterization

OLEDs with active areas of 4 mm^2^ were fabricated by vacuum deposition at ∼10^−5^ Pa on clean ITO-coated glass substrates with a deposition apparatus (SE-4260, ALS Technology, Japan). The deposition rates of HTL, EML, ETL, EIL, and Al were 0.1–0.2, 0.3–0.4, 0.1–0.2, 0.01, and 0.1–0.2 nm s^−1^, respectively. After fabrication, devices were encapsulated with a glass cap using epoxy glue in a N_2_-filled glove box. Calcium oxide was incorporated into the encapsulated package as a desiccant. OLED characteristics were measured with a source meter (2400, Keithley, Japan) and an absolute EQE measurement system (C9920-12, Hamamatsu Photonics, Japan). The integrating sphere for the measurements was calibrated before the experiments.

## Additional information

**How to cite this article:** Kaji, H. *et al*. Purely organic electroluminescent material realizing 100% conversion from electricity to light. *Nat. Commun*. 6:8476 doi: 10.1038/ncomms9476 (2015).

## Supplementary Material

Supplementary Information Supplementary Figures 1-11, Supplementary Tables 1-2, Supplementary Notes 1-9 and Supplementary References

## Figures and Tables

**Table 1:** EQE values for selected DACT-II-*x* devices.

Device	EQE (%)					
	Max	@1 cd m^–2^	@10 cd m^–2^	@100 cd m^–2^	@500 cd m^–2^	@3,000 cd m^–2^
**DACT-II-3**	20.5	18.6	19.4	16.1	13.6	10.1
**DACT-II-6**	28.7	**27.9**	28.1	25.1	21.4	15.3
**DACT-II-9**	**29.6**	23.2	**29.2**	26.5	22.8	16.2
**DACT-II-12**	27.7	21.6	26.6	26.3	23.9	18.0
**DACT-II-19**	27.9	24.8	26.4	**27.9**	**26.9**	**21.8**
**DACT-II-23**	25.2	22.4	23.7	25.2	22.1	17.9
DACT-II-9*	**41.5**	**41.1**	**41.3**	37.9	32.9	24.7
**DACT-II-9**†	40.4	30.7	39.5	**39.9**	**37.6**	**30.7**

EQE, electroluminescence quantum efficiency; DACT-II, 9-[4-(4,6-diphenyl-1,3,5-triazin-2-yl)phenyl]-*N*,*N*,*N*′,*N*′-tetraphenyl-9*H*-carbazole-3,6-diamine.

^*^With out-coupling sheet.

^†^With out-coupling sheet, BmPyPhB was used as the ETL. EQE values in bold are the maximum for respective luminances without and with out-coupling sheet.

**Table 2:** Temperature dependence of various rate constants and PLQYs for CBP films doped with 6 wt% DACT-II.

Temp (K)	*k*_p_ (× 10^8^ s^–1^)	*k*_d_ (× 10^4^ s^–1^)	*k*_ISC_ (× 10^6^ s^–1^)	*k*_RISC_ (× 10^4^ s^–1^)	*Φ*_PL_ (%)	*Φ*_PL_^d^ (%)
200	1.15	5.33	9.18	5.79	97.7	5.7
210	1.16	5.32	9.25	5.79	98.2	6.2
220	1.15	5.39	9.16	5.86	98.4	6.4
230	1.18	5.43	9.39	5.90	98.8	6.8
240	1.15	5.22	9.21	5.68	99.1	7.1
250	1.17	5.52	9.32	5.99	99.6	7.5
260	1.15	5.87	9.21	6.38	99.7	7.7
270	1.16	6.06	9.23	6.58	100.0	8.0
280	1.15	5.75	9.17	6.25	100.0	8.0
290	1.15	6.18	9.17	6.71	100.5	8.4
300	1.16	6.33	9.23	6.88	100.3	8.3

CBP, 4,4′-di(9*H*-carbazol-9-yl)-1,1′-biphenyl; DACT-II, 9-[4-(4,6-diphenyl-1,3,5-triazin-2-yl)phenyl]-*N*,*N*,*N*′,*N*′-tetraphenyl-9*H*-carbazole-3,6-diamine; PLQY, photoluminescence quantum yield; ISC, intersystem crossing; RISC, reverse ISC.

*k*_p_, *k*_d_: radiative rate constants for prompt and delayed components, respectively.

*k*_ISC_, *k*_RISC_: rate constants for ISC and RISC, respectively.

*Φ*_PL_, *Φ*_PL_^d^: PLQYs for total and delayed components, respectively.
