# Supplementary material for: Purely organic electroluminescent material realizing 100% conversion from electricity to light
Source: Nat Commun. 2015 Oct 19;6:8476. doi: 10.1038/ncomms9476 (PMC4634127; doi:10.1038/ncomms9476)
Supplement: Supplementary Information — Supplementary Figures 1-11, Supplementary Tables 1-2, Supplementary Notes 1-9 and Supplementary References [file ncomms9476-s1.pdf]

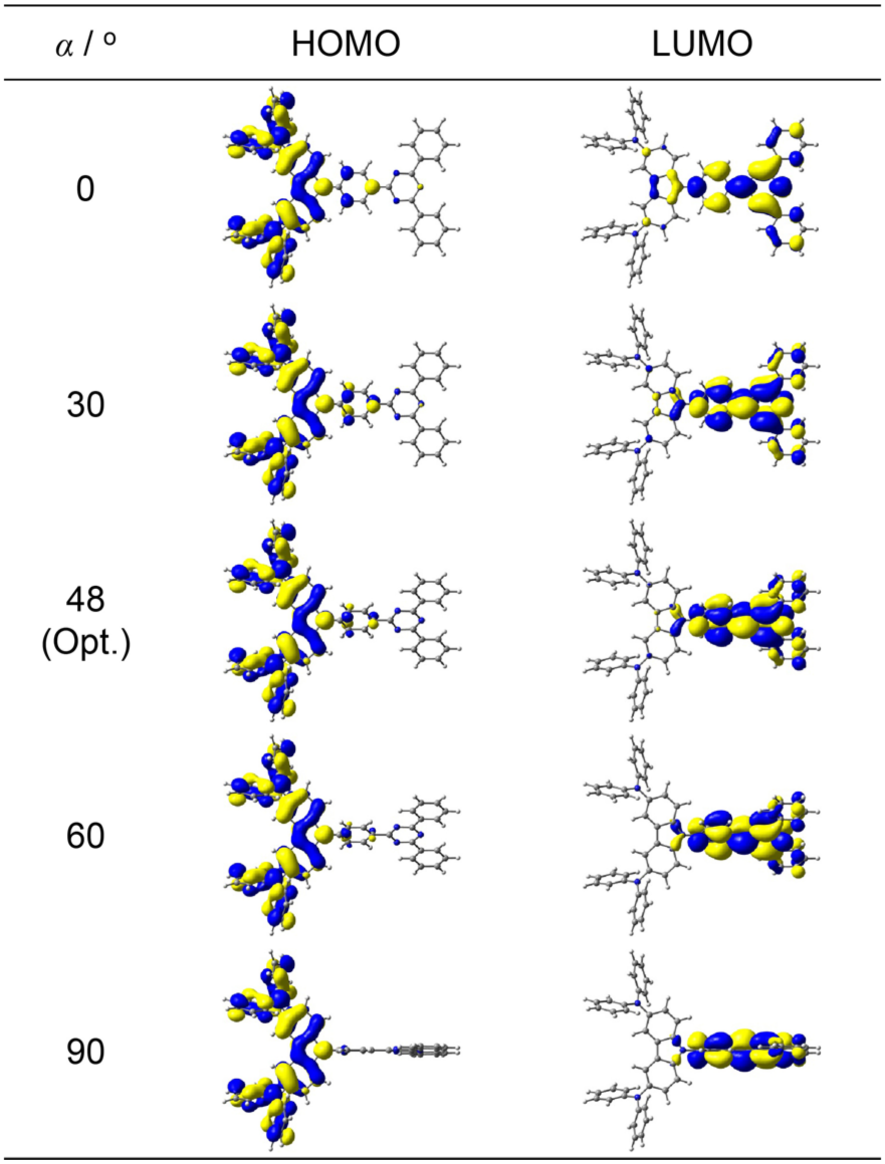

Supplementary Figure 1 | Torsion angle (*α*) dependences of HOMO and LUMO distributions. Calculations were carried out at the PBE0/6-31G(d) level of theory. Structural parameters other than *α* were fixed at their PBE0/6-31G(d)-optimized values.

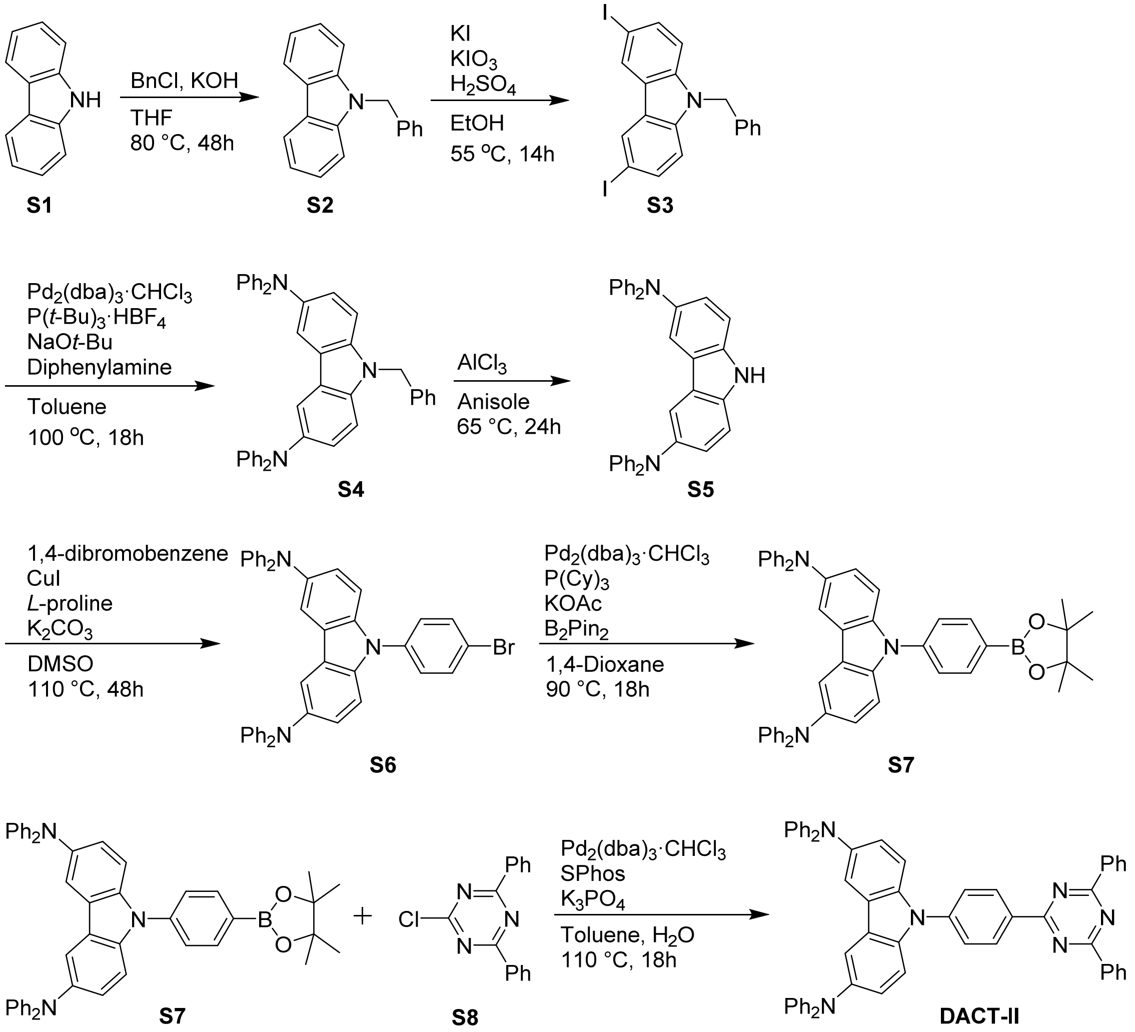

Supplementary Figure 2 | Synthesis of DACT-II.

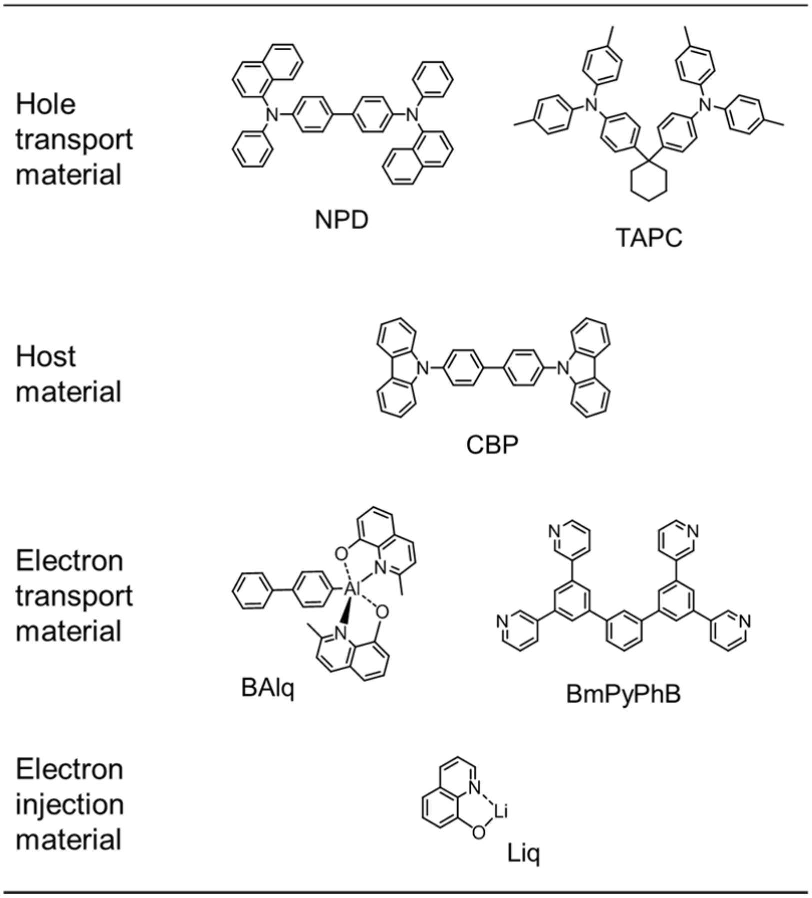

Supplementary Figure 3 | Materials used in OLED fabrication. *N*4,*N*4′-di(naphthalen-1-yl)-*N*4,*N*4′-diphenyl-[1,1′-biphenyl]-4,4′-diamine (NPD) or 4,4′-(cyclohexane-1,1-diyl)bis(*N*,*N*-di-*p*-tolylaniline) (TAPC) was used as the hole-transport layer (HTL), ([1,1′-biphenyl]-4-yloxy)bis((2-methylquinolin-8-yl)oxy)aluminium (BAlq) or 3,3′′,5,5′′-tetra(pyridin-3-yl)-1,1′:3′,1′′-terphenyl (BmPyPhB) as the electron-transport layer (ETL) and lithium quinolin-8-olate (Liq) as the electron-injection layer (EIL). 4,4′-Di(9*H*-carbazol-9-yl)-1,1′-biphenyl (CBP) was used as the host material for the emitter layer (EML).

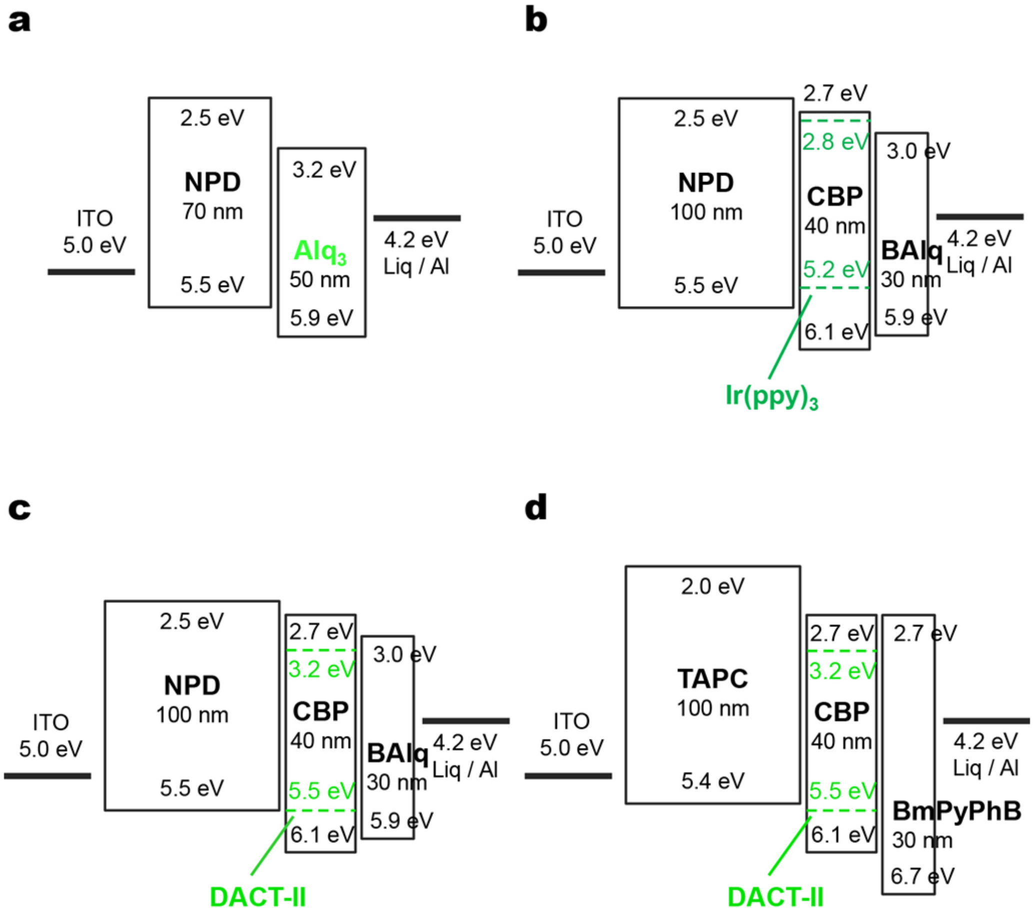

Supplementary Figure 4 | Device structures and energy band diagrams. a, Reference fluorescence OLEDs: ITO/NPD (70 nm)/Alq_3 (50 nm)/Liq/Al (Alq_3 device). **b**, Reference phosphorescence OLEDs: ITO/NPD (100 nm)/CBP:6wt% $Ir(ppy)_3$ (40 nm)/BAlq (30 nm)/Liq/Al ($Ir(ppy)_3$ device). **c**, DACT-II-x OLEDs with NPD as HTL and BAlq as ETL: ITO/NPD (100 nm)/CBP:DACT-II (40 nm)/BAlq (30 nm)/Liq/Al. **d**, DACT-II-x OLEDs with TAPC as HTL and BmPyPhB as ETL: ITO/TAPC (100 nm)/CBP:DACT-II (40 nm)/BmPyPhB (30 nm)/Liq/Al.

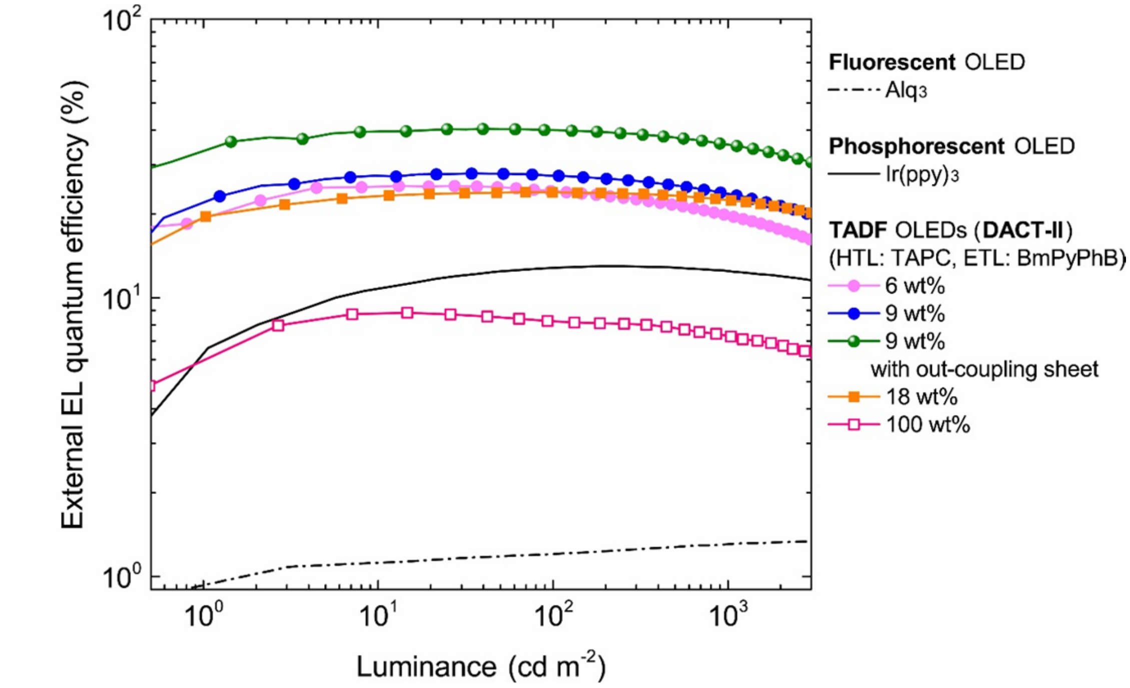

Supplementary Figure 5 | EQE–luminance characteristics of DACT-II-x devices containing BmPyPhB as the ETL. The data for the Alq_3 and $Ir(ppy)_3$ devices are also shown as black dot-dashed and solid lines, respectively. For the DACT-II-100 OLEDs, the EML consisted of DACT-II without CBP.

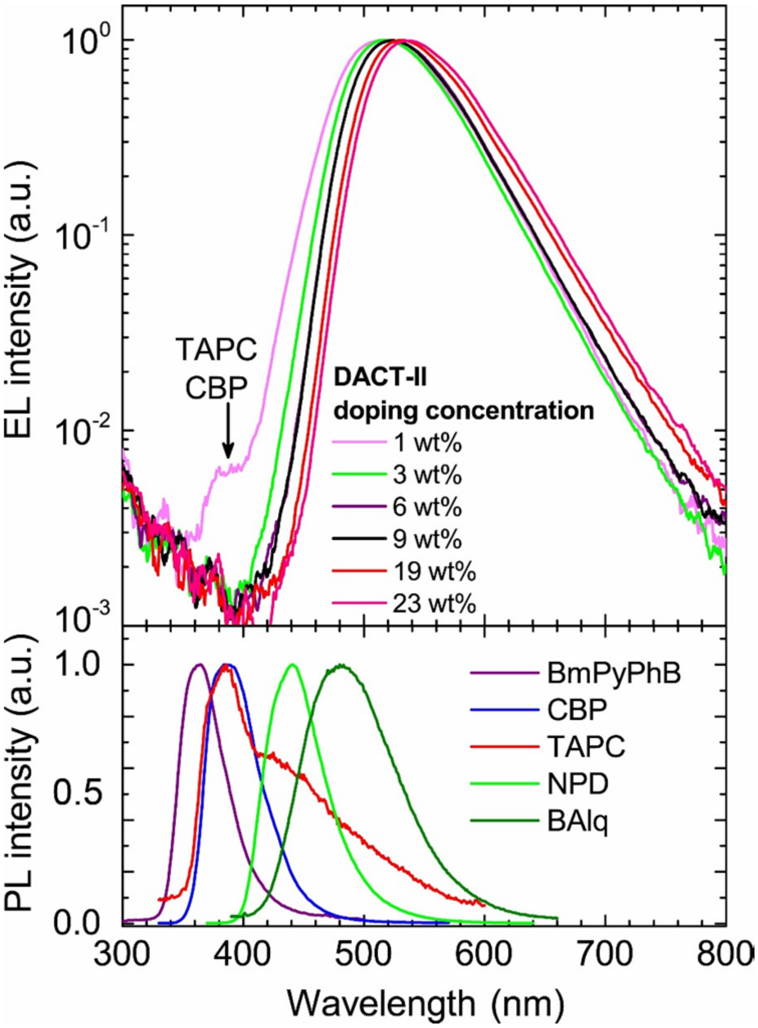

Supplementary Figure 6 | EL spectra (in Fig. 2a) with a logarithmic EL intensity scale. PL spectra of neat films of respective materials used in OLEDs are shown at the bottom for reference.

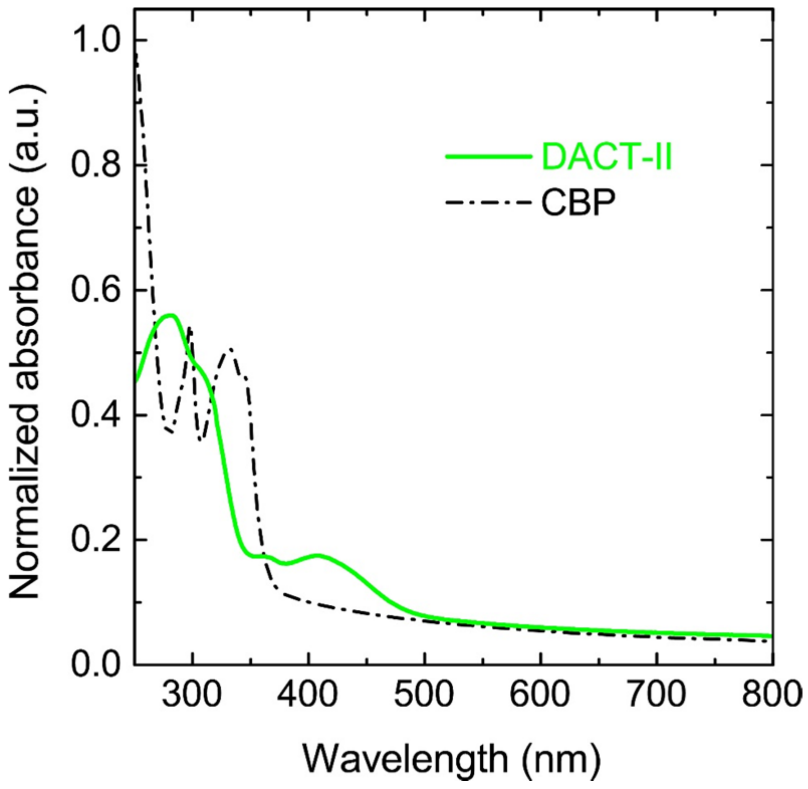

Supplementary Figure 7 | UV-vis spectra of DACT-II and CBP films. The green solid and black dot-dashed lines are UV-vis spectra of DACT-II and CBP films, respectively.

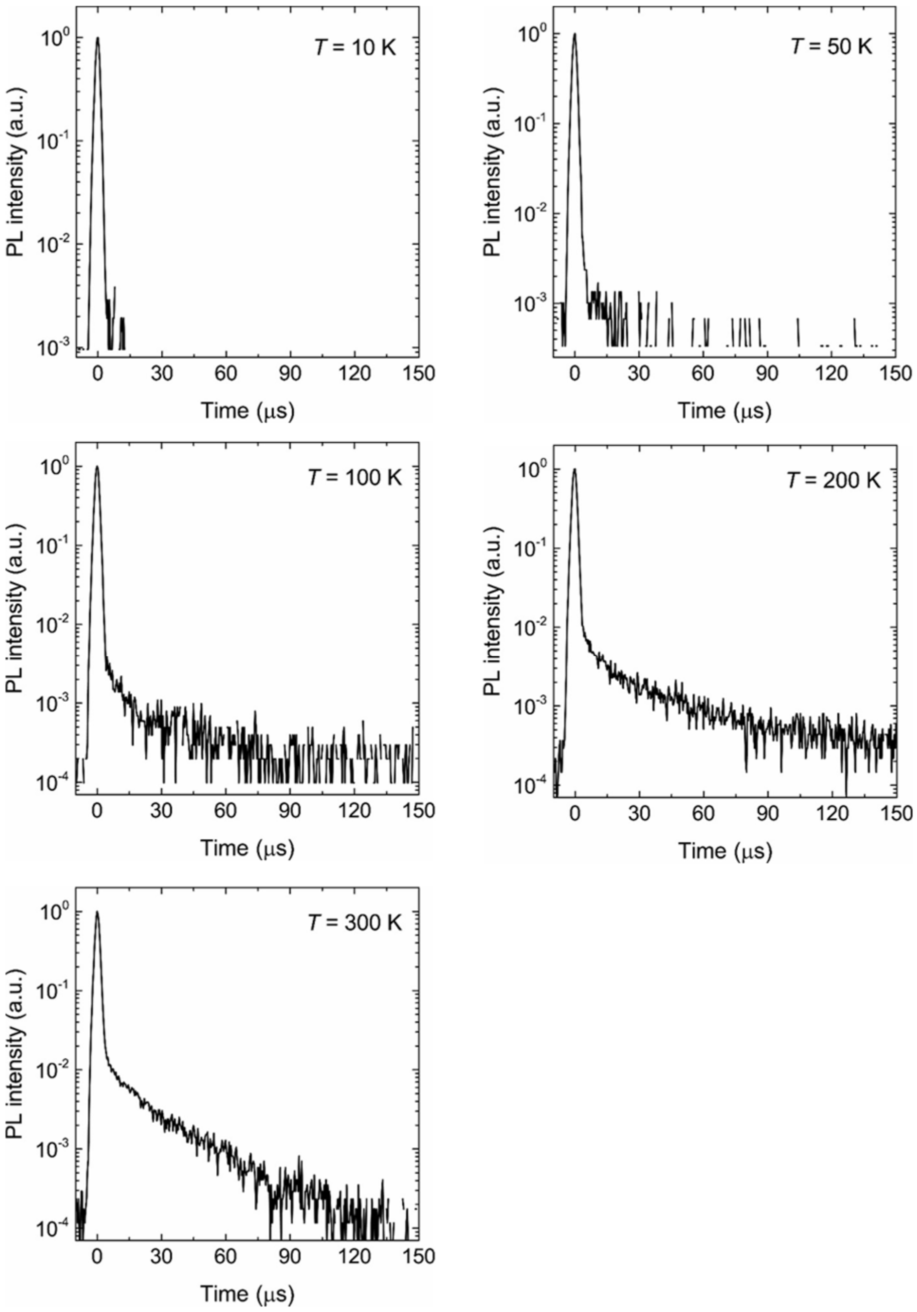

Supplementary Figure 8 | Temperature dependence of the transient PL decay of DACT-II doped into a CBP thin film (6 wt%) from 10 K to 300 K. Some data points are missing, especially for the decay curves at low temperatures. This is because they were negative values and cannot be shown with logarithmic scales.

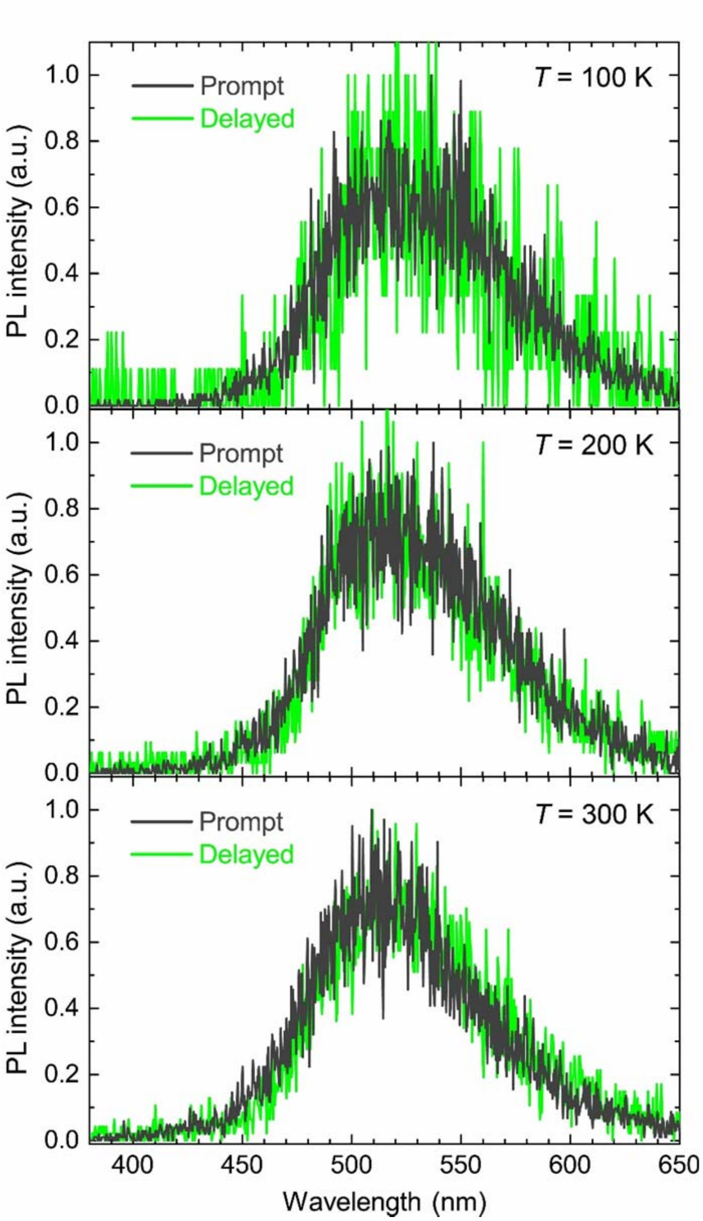

Supplementary Figure 9 | PL emission spectra of the prompt and delayed decay components. Obtained from transient PL measurements at 100, 200 and 300 K.

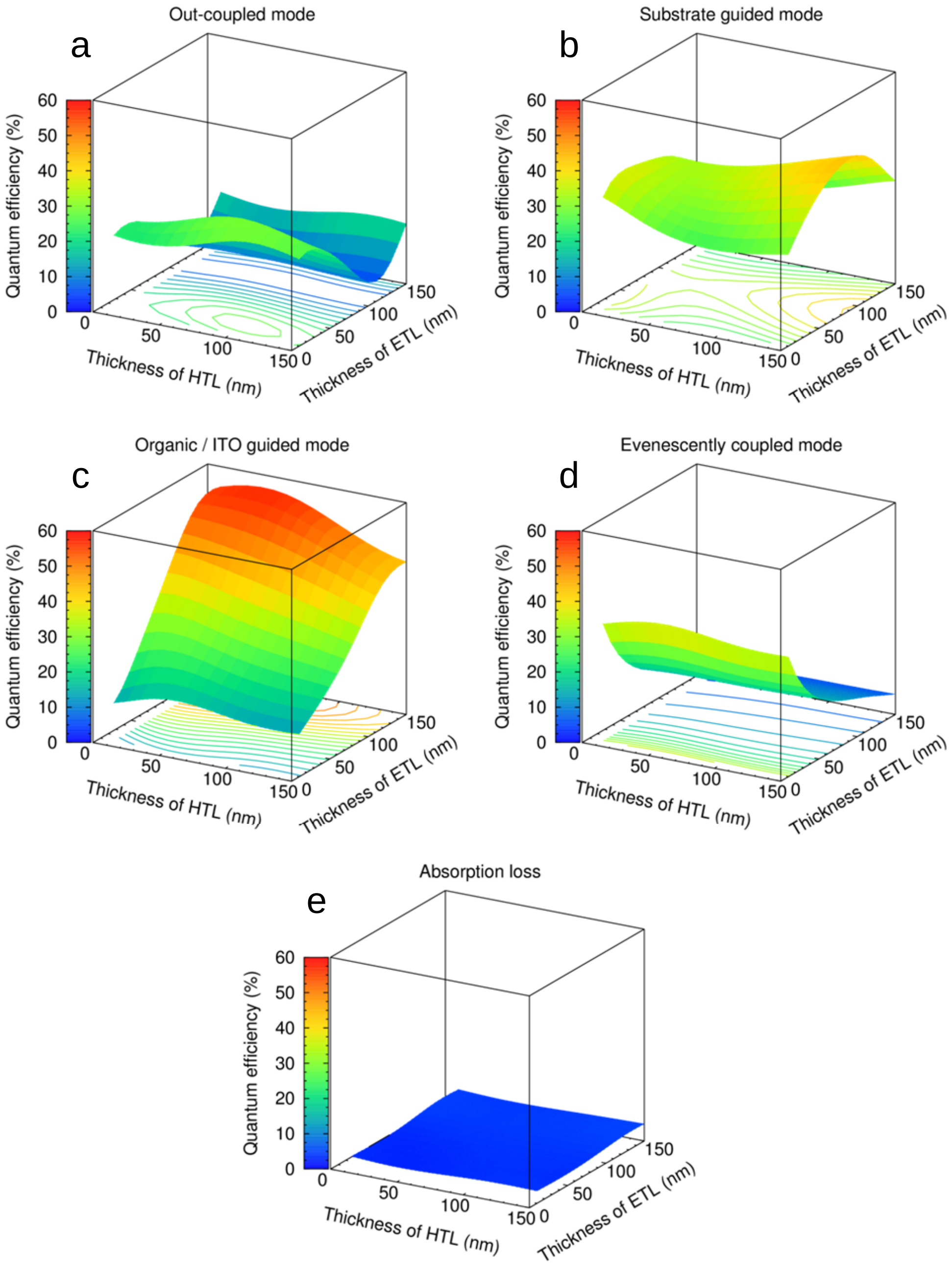

Supplementary Figure 10 | Simulated quantum efficiencies of respective optical modes as a function of HTL and ETL thickness. a, Out-coupled. **b**, Substrate guided. **c**, Organic / ITO guided. **d**, Evanescently coupled. **e**, Absorption loss.

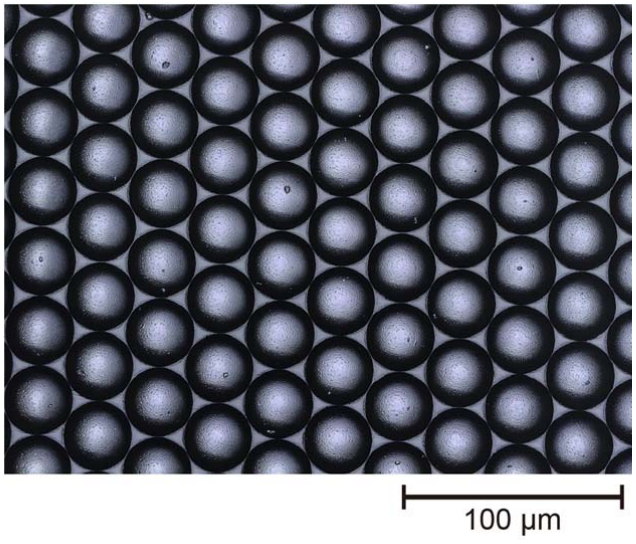

Supplementary Figure 11 | Scanning laser microscope image of the out-coupling sheet with a microlens array used in this study. The diameter and height of the microlens were 29 and 14 μm, respectively.

Supplementary Table 1 | EQE values for OLEDs with the structure ITO/HTL (100 nm)/EML (40 nm)/ETL (30 nm)/Liq/Al *without* out-coupling sheets

Device			EQE (%)						
EML	HTL	ETL	Maximum	@1 cd m^{-2}	@10 cd m^{-2}	@100 cd m^{-2}	@500 cd m^{-2}	@1,000 cd m^{-2}	@3,000 cd m^{-2}
$Ir(ppy)_3$ 6wt%	NPD	BAlq	13.0 @237 cd m^{-2}	6.6	11.0	12.8	12.8	12.5	11.5
DACT-II-**3**	NPD	BAlq	19.9 @3.1 cd m^{-2}	17.7	18.8	15.9	13.5	12.2	10.0
DACT-II-**6**	NPD	BAlq	23.5 @3.4 cd m^{-2}	21.7	23.4	20.6	17.0	15.1	11.7
DACT-II-**1**	TAPC	BAlq	10.5 @3.0 cd m^{-2}	10.9	9.9	9.0	7.8	7.0	5.7
DACT-II-**3**	TAPC	BAlq	20.5 @1.6 cd m^{-2}	18.6	19.4	16.1	13.6	12.4	10.1
DACT-II-**6**	TAPC	BAlq	28.7 @5.1 cd m^{-2}	**27.9**	28.1	25.1	21.4	19.2	15.3
DACT-II-**9**	TAPC	BAlq	**29.6** @4.8 cd m^{-2}	23.2	**29.2**	26.5	22.8	20.4	16.2
DACT-II-**12**	TAPC	BAlq	27.7 @27.8 cd m^{-2}	21.6	26.6	26.3	23.9	21.9	18.0
DACT-II-**19**	TAPC	BAlq	27.9 @93.7 cd m^{-2}	24.8	26.4	**27.9**	**26.9**	**25.3**	**21.8**
DACT-II-**23**	TAPC	BAlq	25.2 @91.1 cd m^{-2}	22.4	23.7	25.2	22.1	20.5	17.9
DACT-II-**6**	TAPC	BmPyPhB	25.2 @27.4 cd m^{-2}	18.5	24.9	24.2	21.6	19.6	16.2
DACT-II-**9**	TAPC	BmPyPhB	27.9 @34.3 cd m^{-2}	23.2	27.5	27.4	25.3	23.6	19.7
DACT-II-**13**	TAPC	BmPyPhB	26.5 @72.5 cd m^{-2}	22.5	25.5	26.4	25.5	24.1	20.9
DACT-II-**18**	TAPC	BmPyPhB	23.9 @69.6 cd m^{-2}	19.6	23.3	23.9	23.2	22.4	20.2
DACT-II-**100**	TAPC	BmPyPhB	8.9 @14.6 cd m^{-2}	4.8	8.7	8.3	7.9	7.3	6.3

Supplementary Table 2 | EQE values for OLEDs with the structure ITO/HTL (100 nm)/EML (40 nm)/ETL (30 nm)/Liq/Al *with* out-coupling sheets

Device				EQE (%)						
Out-coupling Sheet	EML	HTL	ETL	Maximum	@1 cd m^{-2}	@10 cd m^{-2}	@100 cd m^{-2}	@500 cd m^{-2}	@1,000 cd m^{-2}	@3,000 cd m^{-2}
N311-001*	DACT-II-**9**	TAPC	BAlq	**41.5** @7.4 cd m^{-2}	**41.1**	**41.3**	37.9	32.9	30.0	24.7
STC3†	DACT-II-**6**	TAPC	BmPyPhB	30.6 @4.1 cd m^{-2}	35.2	30.5	27.6	23.9	21.8	17.6
STE3†	DACT-II-**6**	TAPC	BmPyPhB	29.0 @8.8 cd m^{-2}	24.3	29.0	26.6	23.1	21.2	17.6
STEP†	DACT-II-**6**	TAPC	BmPyPhB	32.3 @10.5 cd m^{-2}	29.2	32.3	29.3	25.5	23.3	19.4
STEP†	DACT-II-**9**	TAPC	BmPyPhB	37.0 @39.3 cd m^{-2}	31.3	36.2	36.6	34.4	32.0	27.9
130926-A*	DACT-II-**9**	TAPC	BmPyPhB	39.3 @42.3 cd m^{-2}	34.9	37.9	38.8	36.3	34.2	29.5
N311-001*	DACT-II-**9**	TAPC	BmPyPhB	40.4 @39.8 cd m^{-2}	30.7	39.5	**39.9**	**37.6**	**35.5**	**30.7**
130926-A*	DACT-II-**13**	TAPC	BmPyPhB	34.9 @51.9 cd m^{-2}	31.3	34.1	34.7	31.3	32.2	28.8
130926-B*	DACT-II-**13**	TAPC	BmPyPhB	33.5 @107.1 cd m^{-2}	25.8	32.7	33.5	32.6	31.2	27.5
130926-A*	DACT-II-**13**	TAPC	BmPyPhB	33.7 @78.0 cd m^{-2}	23.2	32.5	33.7	32.7	31.3	27.8
N311-001*	DACT-II-**18**	TAPC	BmPyPhB	30.5 @117.8 cd m^{-2}	27.9	29.6	30.5	30.0	29.1	27.0
N331-002*	DACT-II-**18**	TAPC	BmPyPhB	31.1 @230.9 cd m^{-2}	30.3	30.1	31.1	30.8	30.0	27.9

* out-coupling sheets with micro-lens array, containing light-scattering particles

† chemically coated out-coupling sheets containing light-scattering particles

Supplementary Note 1 | Molecular design of a high-performance TADF compound

TADF can be considered a two-step process: $T_1 \rightarrow S_1$ RISC caused by thermal activation and $S_1 \rightarrow S_0$ radiative decay (Fig. 1a). Decreasing ΔE_{ST} increases the rate of $T_1 \rightarrow S_1$ RISC. Meanwhile, increasing the transition dipole moment (μ) for the $S_1 \rightarrow S_0$ transition increases the rate of $S_1 \rightarrow S_0$ radiative decay. ΔE_{ST} is the difference in energy between S_1 at its optimal geometry and T_1 at its optimal geometry. Simultaneously controlling ΔE_{ST} and μ can improve the luminescence efficiency of TADF compounds, because decreasing ΔE_{ST} and increasing μ leads to high luminescence efficiency. Luminescence efficiency is often discussed in terms of oscillator strength *f*. In this study, *f* was calculated by the time-dependent density functional theory and PBE0/6-31G(d) methods. *f* increases with μ, so TADF compounds with high luminescence efficiencies require small ΔE_{ST} and large *f*.

The electronic configurations of S_1 and T_1 were described in terms of singly occupied frontier orbitals. Electrons in molecular orbitals at energies below the HOMO were neglected. This assumption is approximately true for DACT-II because the electronic configurations of its S_1 and T_1 are dominated by the HOMO→LUMO transition. According to the notation of Turro *et al.*[1], the electronic configurations of S_0, S_1 and T_1 can be written as $(\varphi_{HOMO})^2(\varphi_{LUMO})^0$, $(\varphi_{HOMO}\uparrow)^1(\varphi_{LUMO}\downarrow)^1$ and $(\varphi_{HOMO}\uparrow)^1(\varphi_{LUMO}\uparrow)^1$, respectively, where φ_{HOMO} and φ_{LUMO} are the wavefunctions of the HOMO and LUMO, respectively, of a TADF compound in S_0.

Here, we discuss ΔE_{ST} and μ for the same geometry. ΔE_{ST} can then be expressed as

$$\Delta E_{\mathrm{ST}} = 2\left\langle \varphi_{\mathrm{HOMO}}(\mathbf{x}_1)\varphi_{\mathrm{LUMO}}(\mathbf{x}_2) \middle| \frac{1}{|\mathbf{x}_2-\mathbf{x}_1|} \middle| \varphi_{\mathrm{HOMO}}(\mathbf{x}_2)\varphi_{\mathrm{LUMO}}(\mathbf{x}_1) \right\rangle, \qquad (1)$$

where $\mathbf{x}_1$ and $\mathbf{x}_2$ denote the coordinates of electrons in the HOMO and LUMO. μ can be approximately expressed as

$$\mu = \langle \varphi_{\mathrm{HOMO}}(\mathbf{x}) | -e\mathbf{x} | \varphi_{\mathrm{LUMO}}(\mathbf{x}) \rangle, \qquad (2)$$

where $\mathbf{x}$ denotes a point in three-dimensional space. Supplementary Equation (1) shows that ΔE_{ST} is small when the HOMO and LUMO are spatially well separated. Supplementary Equation (2) shows that μ is large when the HOMO and LUMO overlap substantially in a region distant from the origin (*i.e.*, at points where $|\mathbf{x}|$ is large). **The dependence of μ on the HOMO–LUMO overlap differs from that of ΔE_{ST}, so it is possible to increase μ without increasing ΔE_{ST}.** The HOMO and LUMO of DACT-II are spatially well separated, resulting in a small ΔE_{ST}. In addition, a moderate overlap between the HOMO and LUMO sufficiently far from the centre of the molecule leads to a large μ. Precise control of the spatial distribution of the HOMO and LUMO therefore theoretically allows both small ΔE_{ST} and large μ, as shown above. This is experimentally verified for the DACT-II system in this

study.

Supplementary Note 2 | Synthesis of DACT-II

General. ^{1}H and ^{13}C NMR spectra were recorded with a JEOL ECA-600 spectrometer (600 MHz for ^{1}H and 151 MHz for ^{13}C NMR experiments) and Bruker Avance III 800-MHz spectrometer (800 MHz for ^{1}H and 201 MHz for ^{13}C NMR measurements). Chemical shifts are reported in δ ppm, using residual protons in deuterated solvents for ^{1}H NMR experiments and solvent peaks for ^{13}C NMR spectra as internal standards. APCI mass spectra were measured on a Bruker micrOTOF-Q II (Bruker, Germany). All reactions were carried out under Ar atmospheres.

Synthesis

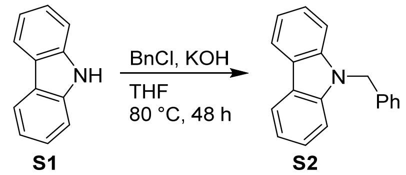

Benzyl chloride (58.4 g, 455 mmol) was added to a mixture of **S1** (25.7 g, 150 mmol) and KOH (41.1 g, 714 mmol) in dry THF (250 mL). The stirred solution was heated under reflux for 48 h. After cooling, the reaction mixture was poured into water and extracted with CH_2Cl_2 (100 mL × 2). The organic phase was dried over anhydrous Na_2SO_4, filtered and concentrated under reduced pressure. The crude product was recrystallized from hexane to

give 30.7 g (119 mmol, 79%) of **S2** as a white solid. ^{1}H NMR (600 MHz, $CDCl_3$): δ 8.15 (d, J = 7.8 Hz, 2H), 7.45 (td, J = 1.2, 7.5 Hz, 2H), 7.38 (d, J = 7.8 Hz, 2H), 7.28–7.25 (m, 5H), 7.16 (d, J = 6.6 Hz, 2H), 5.53 (s, 2H); ^{13}C NMR (151 MHz, $CDCl_3$): δ 148.1, 137.3, 128.9, 127.6, 126.5, 126.0, 123.2, 120.5, 119.3, 109.0, 46.70.

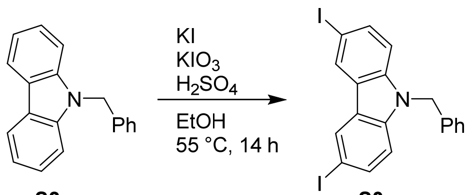

S2 **S3**

Concentrated sulfuric acid (3.07 g, 31.3 mmol) was added to a suspension of **S2** (5.1 g, 19.8 mmol), KIO_3 (3.34 g, 16.9 mmol) and KI (5.38 g, 32.4 mmol) in ethanol (700 mL). The reaction mixture was stirred at 55 °C for 14 h in the dark. The reaction mixture was poured into aqueous sodium bisulphite solution and extracted with dichloromethane (100 mL × 3). The combined organic extracts were washed with water, dried over anhydrous Na_2SO_4, filtered and evaporated *in vacuo*. The crude product was recrystallized from ethyl acetate/hexane to give 9.65 g (19.0 mmol, 96%) of **S3** as a white solid. ^{1}H NMR (600 MHz, $CDCl_3$): δ 8.37 (d, J = 1.8 Hz, 2H), 7.68 (dd, J = 1.8, 8.7 Hz, 2H), 7.27–7.24 (m, 3H), 7.13 (d, J = 8.4 Hz, 2H), 7.05 (dd, J = 1.8, 7.8 Hz, 2H), 5.45 (s, 2H); ^{13}C NMR (151 MHz, $CDCl_3$): δ 139.8, 136.3, 134.9, 129.5, 129.1, 127.9, 126.4, 124.3, 11.3, 109.0, 46.8; APCI-MS (m/z): $[M]^+$ calcd. for $C_{19}H_{13}I_2N$, 508.9137; found, 508.9127.

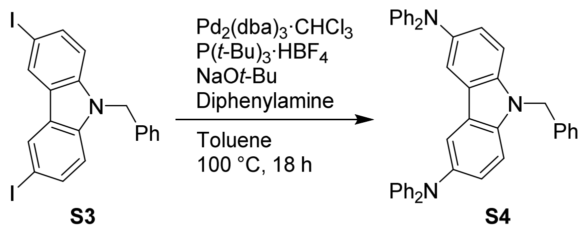

S3 (4.30 g, 8.45 mmol), $Pd_2(dba)_3 \cdot CHCl_3$ (56.0 mg, 54.1 μmol), $P(t\text{-Bu})_3 \cdot HBF_4$ (62.7 mg, 0.219 μmol), NaO*t*-Bu (2.80 g, 29.1 mmol) and diphenylamine (3.21 g, 19.0 mmol) were dissolved in dry toluene (200 mL). The mixture was stirred at 100 °C for 18 h. After cooling, the reaction mixture was poured into water and extracted with toluene (120 mL × 3). The organic phase was dried over anhydrous Na_2SO_4, filtered and concentrated under reduced pressure. The crude product was purified by silica gel column chromatography (CH_2Cl_2:hexane = 1:4, R_f = 0.33) to give 4.24 g (7.17 mmol, 84%) of **S4** as a pale-green solid. ^{1}H NMR (600 MHz, $CDCl_3$): δ 7.76 (s, 2H), 7.33 (t, J = 7.2 Hz, 2H), 7.29–7.28 (m, 3H), 7.24–7.22 (m, 4H), 7.19 (t, J = 7.8 Hz, 8H), 7.06 (d, J = 7.8 Hz, 8H), 6.92 (t, J = 7.2 Hz, 4H), 5.47 (s, 2H); ^{13}C NMR (151 MHz, $CDCl_3$): δ 148.7, 140.1, 138.6, 137.3, 129.2, 129.0, 127.8, 126.7, 126.1, 123.8, 122.8, 121.7, 119.0, 110.0, 47.1; APCI-MS (*m/z*): $[M+H]^+$ calcd. for $C_{43}H_{34}N_3$, 592.2753; found, 592.2752.

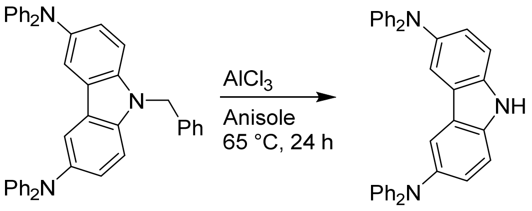

S4 (3.06 g, 5.18 mmol) in dry anisole (16 mL) was added to a suspension of $AlCl_3$ (5.04 g, 37.8 mmol) in dry anisole (5.0 mL) at 0 °C. The reaction mixture was stirred at 65 °C for 24 h. The reaction mixture was poured into water and extracted with toluene (80 mL × 2). The organic phase was dried over anhydrous Na_2SO_4, filtered and concentrated under reduced pressure. The crude product was purified by silica gel column chromatography (CH_2Cl_2:hexane = 1:1, R_f = 0.38) to give 2.23 g (4.45 mmol, 86%) of **S5** as a pale-green solid. ^1H NMR (600 MHz, CD_2Cl_2): δ 8.13 (s, 1H), 7.70 (d, J = 1.8 Hz, 2H), 7.41 (d, J = 9.0 Hz, 2H), 7.23–7.16 (m, 10H), 7.03 (d, J = 8.4 Hz, 8H), 6.93 (t, J = 7.2 Hz, 4H); ^{13}C NMR (151 MHz, CD_2Cl_2): δ 149.0, 140.4, 137.8, 129.4, 126.4, 124.5, 123.0, 120.0, 119.1, 112.2; APCI-MS (m/z): $[M+H]^+$ calcd. for $C_{36}H_{28}N_3$, 502.2283; found, 502.2256.

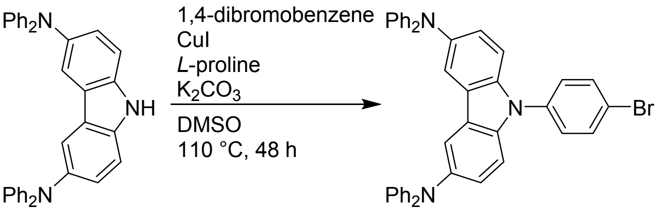

S5 (2.00 g, 4.00 mmol), 1,4-dibromobenzene (1.41 g, 6.01 mmol), CuI (75.9 mg, 0.40 mmol), L-proline (92.1 mg, 0.80 mmol) and K_2CO_3 (1.12 g, 8.01 mmol) were mixed in dry

dimethyl sulfoxide (6.0 mL) and stirred at 110 °C for 48 h. The reaction mixture was poured into water and extracted with CH_2Cl_2 (75 mL × 3). The organic phase was dried over anhydrous Na_2SO_4, filtered, and concentrated under reduced pressure. The crude product was purified by silica gel column chromatography (CH_2Cl_2:hexane = 1:4, R_f = 0.43) to give 1.92 g (2.93 mmol, 73%) of **S6** as a pale-green solid. ^{1}H NMR (600 MHz, $CDCl_3$): δ 7.73 (d, J = 8.4 Hz, 2H), 7.47 (d, J = 9.0 Hz, 2H), 7.30 (d, J = 9.0 Hz, 2H), 7.22–7.19 (m, 10H), 7.06 (d, J = 7.8 Hz, 8H), 6.94 (t, J = 7.20 Hz, 4H); ^{13}C NMR (151 MHz, $CDCl_3$): δ 148.6, 141.0, 138.2, 136.8, 133.3, 129.2, 128.6, 126.1, 124.3, 122.9, 121.9, 121.0 118.8, 110.8; APCI-MS (*m/z*): $[M+H]^+$ calcd. for $C_{42}H_{31}BrN_3$, 656.1701; found, 656.1673.

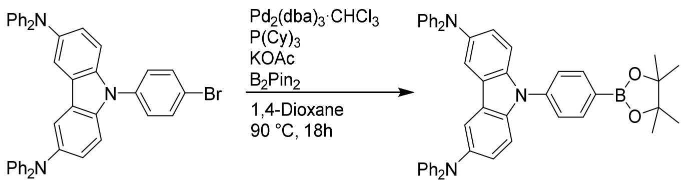

S6 **S7**

$Pd_2(dba)_3 \cdot CHCl_3$ (56.0 mg, 54.1 μmol) and $P(Cy)_3$ (63.8 mg, 227 μmol) were dissolved in 1,4-dioxane (5 mL) and stirred for 30 min at room temperature. **S6** (1.60 g, 2.44 mmol), B_2Pin_2 (0.68 g, 2.68 mmol) and KOAc (0.48 g, 4.88 mmol) were added to the above mixture, which was then stirred at 90 °C for 18 h. After cooling, the reaction mixture was poured into water and extracted with toluene (60 mL × 3). The organic phase was washed with brine, dried over anhydrous Na_2SO_4, filtered and concentrated under reduced pressure to give 1.81

g of crude **S7** as a brown solid. The crude product was used for the next step without further purification. APCI-MS (*m/z*): $[M+H]^+$ calcd. for $C_{48}H_{43}BN_3O_2$, 704.3448; found, 704.3432.

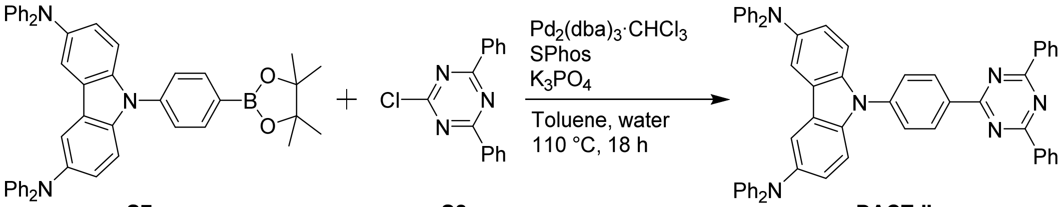

Crude **S7** (1.81 g), **S8** (0.78 g, 2.93 mmol), $Pd_2(dba)_3 \cdot CHCl_3$ (37.9 mg, 36.6 μmol), SPhos (60.8 mg, 148 μmol) and K_3PO_4 (1.04 g, 4.89 mmol) were suspended in toluene (3 mL) and water (0.1 mL), and stirred at 110 °C for 18 h. After cooling, the reaction mixture was poured into water and extracted with CH_2Cl_2. The organic phase was dried over anhydrous Na_2SO_4, filtered and concentrated under reduced pressure. The crude product was purified by silica gel column chromatography (CH_2Cl_2:hexane = 1:4, R_f = 0.26) and recrystallized from CH_2Cl_2/hexane to give 1.26 g (1.56 mmol, 64% from **S6**) of **DACT-II** as a yellow solid. ^1H NMR (800 MHz, CD_2Cl_2): δ 9.04 (d, J = 8.6Hz, 2H), 8.83 (d, J = 8.2 Hz, 4H), 7.86 (d, J = 8.6 Hz, 2H), 7.781 (d, J = 2.1 Hz, 2H), 7.67 (t, J = 7.2 Hz, 2H), 7.63 (t, J = 7.3 Hz, 4H), 7.53 (d, J = 8.7 Hz, 2H), 7.26 (dd, J = 2.2, 8.8 Hz, 2H), 7.22 (t, J = 8.0 Hz, 8H), 7.07 (d, J = 8.1 Hz, 8H), 6.95 (t, J = 7.3 Hz, 4H); ^{13}C NMR (201 MHz, CD_2Cl_2): δ 172.3, 171.4, 149.0, 142.0, 141.5, 138.4, 136.6, 135.3, 133.1, 131.1, 129.5, 129.4, 129.2, 126.9, 126.3, 125.0, 123.3, 122.2, 118.9, 111.5; APCI-MS (*m/z*): $[M+H]^+$ calcd. for $C_{57}H_{41}N_6$, 809.3393;

found, 809.3341; elemental analysis calcd. (%) for $C_{57}H_{40}N_6$: C 84.63, H 4.98, N 10.39; found: C 84.76, H 4.79, N 10.32.

Supplementary Note 3 | Thermophysical characterization

Glass transition, crystallization and melting temperatures of a powder sample of **DACT-II** were measured by differential scanning calorimetry (DSC1, Mettler Toledo, Switzerland). Glass transition temperatures of amorphous thin film samples with a thickness of 100 nm were measured by ultrafast differential scanning calorimetry (Flash DSC, Mettler Toledo). The film samples were deposited directly on chip sensors. Decomposition and sublimation temperatures of powder samples of **DACT-II** corresponded to 5% weight loss (under atmospheric pressure and ~20 Pa, respectively) in thermogravimetric analysis (TG-DTA2000SE.S.25.3, Netzsch, Japan).

Supplementary Note 4 | Origin of red shifts in PL and EL spectra

As shown in Fig. 2a and b, small red shifts were observed in PL and EL spectra with increasing DACT-II concentration. DACT-II is composed of donor and acceptor segments and has large dipole moment. The dipole moment of the host molecule CBP is small. Therefore, the origin of the red shift observed in PL experiments is considered to be the increase of polarity of the matrix with increasing DACT-II concentration. If we consider a

single DACT-II molecule, the polarity of the surrounding matrix increases with DACT-II concentration because the matrix is composed of a mixture of other DACT-II molecules and CBP. The polar matrix stabilizes the excited state of DACT-II and induces the red shift of emission spectrum, as reported by Bulović *et al.*[2]. In EL experiments, the position of the hole–electron recombination zone may also slightly change with doping concentration. This will in turn modify the emission wavelength.

Supplementary Note 5 | Photophysical characterization

Solutions of **DACT-II** with a concentration of 1.0×10^{-5} mol L^{-1} were prepared using toluene as a solvent. Organic films were fabricated by vacuum deposition at ~10^{-5} Pa on clean quartz glass substrates. Ultraviolet–visible (UV–vis) absorption and PL spectra were measured with UV–vis (UV-3150, Shimadzu, Japan) and PL (Quantaurus-QY C11347-01 Hamamatsu Photonics, Japan and FluoroMax-4, Horiba Scientific, Japan) spectrometers, respectively. The excitation wavelengths used for PL spectra measurements were 340, 400, and 405 nm. Emission lifetimes were measured using a fluorescence lifetime measurement system (Quantaurus-Tau C11367-02, Hamamatsu Photonics). In emission lifetime experiments, the excitation wavelength was 405 nm for both the solution and neat film samples. We used excitation wavelengths of 340 and 405 nm, which mainly excited CBP and DACT-II, respectively, for the doped film samples. We confirmed that emission lifetime did

not depend on the excitation wavelength for the doped film samples under our experimental conditions, indicating that the energy transfer from CBP to DACT-II is very fast compared to prompt and delayed radiative decays. The values of Φ_{PL} were measured using an absolute PL quantum yield measurement system (Quantaurus-QY C11347-01, Hamamatsu Photonics). In these experiments, solutions of DACT-II were directly excited at a wavelength of 400 nm. For the film samples, we used excitation wavelengths of 340 and 405 nm, where the CBP host and DACT-II were mainly excited, respectively. The PLQYs for the 9 wt% DACT-II-doped CBP thin films were 100% (within the experimental error) irrespective of the excitation wavelength, indicating complete energy transfer from CBP to DACT-II. Low-temperature PL intensity and emission lifetime were measured using a streak camera (C4334, Hamamatsu Photonics). A 337-nm N_2 gas laser (KEC-X, USHO, Japan) was used as the excitation source. Decay curves were measured on scales of nanoseconds and microseconds for the prompt and delayed components, respectively. Ionization potentials (Ip) were measured for the neat film samples with an atmospheric photoelectron spectrophotometer (AC-3, RIKEN KEIKI, Japan). Electron affinity (Ea) was estimated from the Ip and optical band gap. The determined Ip and Ea are shown in Fig. 1d and Supplementary Fig. 4.

Supplementary Note 6 | Analysis of k_{RISC} and ΔE_{ST}

The time dependence of the singlet concentration $[S_1]$ and triplet concentration $[T_1]$ is described by

$$\frac{d}{dt}\begin{pmatrix}[S_1]\\ [T_1]\end{pmatrix} = \begin{pmatrix}-(k_{\mathrm{r}}^{\mathrm{S}} + k_{\mathrm{nr}}^{\mathrm{S}} + k_{\mathrm{ISC}}) & k_{\mathrm{RISC}} \\ k_{\mathrm{ISC}} & -(k_{\mathrm{r}}^{\mathrm{T}} + k_{\mathrm{nr}}^{\mathrm{T}} + k_{\mathrm{RISC}})\end{pmatrix}\begin{pmatrix}[S_1]\\ [T_1]\end{pmatrix}, \quad (3)$$

where $k_{\mathrm{r}}^{\mathrm{S}}$ and $k_{\mathrm{nr}}^{\mathrm{S}}$ are the rate constants for radiative and non-radiative decay from S_1, respectively, $k_{\mathrm{r}}^{\mathrm{T}}$ and $k_{\mathrm{nr}}^{\mathrm{T}}$ are the rate constants of radiative and non-radiative decay from T_1, respectively, and k_{ISC} and k_{RISC} are the rate constants for ISC and RISC, respectively. The solution of Supplementary Equation (3) is then

$$\begin{pmatrix}[S_1]\\ [T_1]\end{pmatrix} = \begin{pmatrix}p_1 C_1 \exp(\lambda_1 t) + q_1 C_2 \exp(\lambda_2 t)\\ p_2 C_1 \exp(\lambda_1 t) + q_2 C_2 \exp(\lambda_2 t)\end{pmatrix}, \quad (4)$$

where p_1, p_2, q_1, q_2, C_1 and C_2 are prefactors. λ_1 and λ_2 are the solution to the following equation

$$\{(k_{\mathrm{r}}^{\mathrm{S}} + k_{\mathrm{nr}}^{\mathrm{S}} + k_{\mathrm{ISC}}) + \lambda\}\{(k_{\mathrm{r}}^{\mathrm{T}} + k_{\mathrm{nr}}^{\mathrm{T}} + k_{\mathrm{RISC}}) + \lambda\} - k_{\mathrm{ISC}}k_{\mathrm{RISC}} = 0. \quad (5)$$

The time dependence of the observable $[S_1]$ is provided by double exponentials according to

$$[S_1] = p_1 C_1 \exp(\lambda_1 t) + q_1 C_2 \exp(\lambda_2 t) = I_\mathrm{p} \exp(-k_\mathrm{p} t) + I_\mathrm{d} \exp(-k_\mathrm{d} t), \quad (6)$$

where I_p and k_p are the intensity and rate constant, respectively, of the prompt emission component, while I_d and k_d are the respective intensity and rate constant the delayed emission component. These can be obtained experimentally from PL (or EL) decay measurements.

Here, $-k_\mathrm{p}$ and $-k_\mathrm{d}$ are the solutions of Supplementary Equation (6), and thus

$$\{(k_\mathrm{r}^\mathrm{S} + k_\mathrm{nr}^\mathrm{S} + k_\mathrm{ISC}) - k_\mathrm{p}\}\{(k_\mathrm{r}^\mathrm{T} + k_\mathrm{nr}^\mathrm{T} + k_\mathrm{RISC}) - k_\mathrm{p}\} - k_\mathrm{ISC} k_\mathrm{RISC} = 0, \quad (7)$$

$$\{(k_\mathrm{r}^\mathrm{S} + k_\mathrm{nr}^\mathrm{S} + k_\mathrm{ISC}) - k_\mathrm{d}\}\{(k_\mathrm{r}^\mathrm{T} + k_\mathrm{nr}^\mathrm{T} + k_\mathrm{RISC}) - k_\mathrm{d}\} - k_\mathrm{ISC} k_\mathrm{RISC} = 0. \quad (8)$$

When $k_\mathrm{p} \neq k_\mathrm{d}$, the following equations are obtained by subtracting and adding Supplementary Equations (7) and (8):

$$k_\mathrm{p} + k_\mathrm{d} = k_\mathrm{r}^\mathrm{S} + k_\mathrm{nr}^\mathrm{S} + k_\mathrm{ISC} + k_\mathrm{r}^\mathrm{T} + k_\mathrm{nr}^\mathrm{T} + k_\mathrm{RISC}, \quad (9)$$

$$k_\mathrm{p} k_\mathrm{d} = (k_\mathrm{r}^\mathrm{S} + k_\mathrm{nr}^\mathrm{S} + k_\mathrm{ISC})(k_\mathrm{r}^\mathrm{T} + k_\mathrm{nr}^\mathrm{T} + k_\mathrm{RISC}) - k_\mathrm{ISC} k_\mathrm{RISC}. \quad (10)$$

The total PLQY (Φ_PL) is the sum of the prompt (${\Phi_\mathrm{PL}}^\mathrm{p}$) and delayed (${\Phi_\mathrm{PL}}^\mathrm{d}$) components, and

the experimental Φ_{PL} of the DACT-II-doped CBP films is approximately 100% (Table 2). Phosphorescence was not observed at any temperature. Therefore, it is reasonably assumed that 1) $k_{\mathrm{r}}^{\mathrm{S}} \gg k_{\mathrm{nr}}^{\mathrm{S}}, k_{\mathrm{r}}^{\mathrm{T}}, k_{\mathrm{nr}}^{\mathrm{T}}$ and 2) $k_{\mathrm{RISC}} \gg k_{\mathrm{r}}^{\mathrm{T}}, k_{\mathrm{nr}}^{\mathrm{T}}$. It is experimentally observed that $k_{\mathrm{p}} \gg k_{\mathrm{d}}$ (Table 2). Supplementary Equations (9) and (10) can then be reduced to

$$k_{\mathrm{p}} \approx k_{\mathrm{r}}^{\mathrm{S}} + k_{\mathrm{ISC}} + k_{\mathrm{RISC}}, \tag{11}$$

$$k_{\mathrm{p}} k_{\mathrm{d}} \approx k_{\mathrm{r}}^{\mathrm{S}} k_{\mathrm{RISC}}. \tag{12}$$

Previous studies of TADF OLEDs have generally assumed that $k_{\mathrm{ISC}} \gg k_{\mathrm{RISC}}$. This cannot be readily assumed here because ΔE_{ST} is much smaller than that in previous studies. From Supplementary Equations (11) and (12),

$$k_{\mathrm{RISC}} \approx \frac{1}{2}\left(k_{\mathrm{p}} - k_{\mathrm{ISC}} \pm \sqrt{\left(k_{\mathrm{p}} - k_{\mathrm{ISC}}\right)^2 - 4 k_{\mathrm{p}} k_{\mathrm{d}}}\right). \tag{13}$$

From Supplementary Equation (13) and the experimentally obtained k_{p} (~10^8 s^{-1}), k_{d} (~10^4 s^{-1}) and k_{ISC} (~10^7 s^{-1}), k_{RISC} of approximately 10^4–10^5 s^{-1} is obtained. This k_{RISC} is larger than that for 4CzIPN (~10^3 s^{-1} for ΔE_{ST} = 82.6 meV). However, the assumption of $k_{\mathrm{ISC}} \gg k_{\mathrm{RISC}}$ is still reasonable for DACT-II systems. The other solution of Supplementary Equation (13) is ~10^8 s^{-1}, which is physically unreasonable because $k_{\mathrm{RISC}} > k_{\mathrm{ISC}}$. We then have

$$k_{\mathrm{p}} \approx k_{\mathrm{r}}^{\mathrm{S}} + k_{\mathrm{ISC}}, \tag{14}$$

$$k_{\mathrm{RISC}} \approx \frac{k_{\mathrm{p}} k_{\mathrm{d}}}{k_{\mathrm{p}} - k_{\mathrm{ISC}}}. \tag{15}$$

The PLQY is obtained from Supplementary Equation (14) as

$$\Phi_{\mathrm{PL}}{}^{\mathrm{p}} \approx \frac{k_{\mathrm{r}}^{\mathrm{S}}}{k_{\mathrm{r}}^{\mathrm{S}} + k_{\mathrm{ISC}}} \approx \frac{k_{\mathrm{r}}^{\mathrm{S}}}{k_{\mathrm{p}}}. \tag{16}$$

$\Phi_{\mathrm{PL}}{}^{\mathrm{p}}$ and $\Phi_{\mathrm{PL}}{}^{\mathrm{d}}$ can be calculated from the experimentally obtained values of $I_{\mathrm{p}}, k_{\mathrm{p}}, I_{\mathrm{d}}, k_{\mathrm{d}}$ and Φ_{PL} (see Supplementary Equation (6)).

From (14) and (16), we can calculate k_{ISC} as

$$k_{\mathrm{ISC}} \approx k_{\mathrm{p}} - k_{\mathrm{r}}^{\mathrm{S}} = k_{\mathrm{p}}(1 - \Phi_{\mathrm{PL}}{}^{\mathrm{p}}). \tag{17}$$

The activation energy ΔE_{ST} is obtained from[3,4]

$$k_{\mathrm{RISC}} = A\, exp\left(-\frac{\Delta E_{\mathrm{ST}}}{k_{\mathrm{B}} T}\right), \tag{18}$$

where A is a prefactor.

Supplementary Note 7 | Analysis of transition dipole moment orientation

Variable angle spectroscopic ellipsometry (VASE) of a neat film of DACT-II on a Si substrate was measured with a spectroscopic ellipsometer (M-2000U, J. A. Woollam Co. Inc.). Incident angles were varied from 45° to 75° with steps of 5°. Anisotropic optical parameters, extinction coefficient k and refractive index n, for the film were obtained by analysing the experimental ellipsometric parameters Ψ and Δ using a uniaxial anisotropy model with Tauc-Lorentz-type and Gaussian-type oscillators *via* WVASE32 software (J. A. Woollam Co. Inc.).

Using the ordinary and extraordinary extinction coefficients, k_o and k_e, respectively, at 411 nm, which corresponds to the electronic absorption band for the transition dipole moment parallel to the longest axis of DACT-II, the orientation order parameter, S, of DACT-II is calculated to be S = -0.32 ± 0.01 from the following equation[5],

$$S = \frac{1}{2}(3\cos^2\theta - 1) = \frac{k_e - k_o}{k_e + 2k_o}, \tag{19}$$

where θ is the angle between the normal of the substrate and transition dipole.

Angular-dependent PL experiments were also carried out to analyse the orientation

of transition dipole moment of DACT-II in vacuum-deposited thin films. The dipole orientation for DACT-II 9 wt%-doped CBP film on a quartz substrate was analysed by the dependence of light emission intensity in transverse magnetic mode on the emission angle with a system composed of a 375-nm continuous-wave diode laser (DPS-5004, Neoarc), a rotation stage, a polarized filter, and a calibrated multichannel spectrometer (PMA-12, Hamamatsu Photonics) combined with a collimator and optical fiber. PL intensities at 515 nm were monitored while rotating the stage at a fixed excitation angle of 45°. A fused silica half-cylinder prism was attached to the quartz substrate using matching liquid. The analysis of the experimental data using Setfos software (FLUXiM Inc., version 3.4) provided a direction cosine of the transition dipole moment along the normal direction to the substrate, α, of 0.22 ± 0.02. This value corresponds to $p_z : p_x = 0.56 \pm 0.07 : 1$ where p_z and p_x are transition dipole moments perpendicular and parallel to the substrate, respectively, and S of -0.29 ± 0.05, according to the following equations[6].

$$\frac{p_z}{p_x} = \frac{2\alpha}{1-\alpha}, \tag{20}$$

$$S = \frac{4\alpha^2-(1-\alpha)^2}{4\alpha^2+2(1-\alpha)^2} = \frac{{p_z}^2-{p_x}^2}{{p_z}^2+2{p_x}^2}. \tag{21}$$

In the main text, we used the order parameter, S, to describe orientation, because the

parameter is most commonly used for the quantitative description of orientation.

Supplementary Note 8 | Electrical and optical simulations

Electrical and optical simulations were carried out using Setfos software (version 4.1). Device structure used in the simulation was glass substrate (0.7 mm) / ITO (50 nm) / TAPC / CBP: DACT-II (40 nm) / BAlq / Al (80 nm). The thicknesses of TAPC and BAlq layers were varied from 10 to 150 nm in intervals of 10 nm. The electron injection layer (Liq) was ignored in the simulations because it was only 1 nm thick. The location of the recombination zone was obtained from the electrical simulations, which was then used as the emission zone for the optical simulations. EQEs were calculated by integrating the quantum efficiency of the out-coupled mode with respect to emission wavelength with weighting of the intensity distribution of PL spectra. Maximum EQEs were obtained for the devices containing TAPC and BAlq layers with thicknesses of 100 and 30 nm, respectively. On the basis of VASE and angular-dependent PL experiments, which gave S = -0.32 ± 0.01 and -0.29 ± 0.05, respectively, EQEs of 29.5 ± 0.2% and 29.0 ± 0.9% were obtained. Both of these values are in good agreement with the experimental maximum EQE of 29.6%. Assumption of randomly oriented transition dipole moments with $S = 0$ gave an EQE of 24.2%.

VASE experiments observe transition dipole moment for UV-vis absorption. In contrast, angular-dependent PL experiments observe transition dipole moment for PL

emission. In the main text, *S* obtained from angular-dependent PL experiments are used.

Supplementary Note 9 | Scanning laser microscopy

The surface image of the out-coupling sheet used in this study was recorded by a shape analysis laser microscope (VK-X250/260, Keyence, Japan).

Supplementary References

1. Turro, N. J., Ramamurthy, V. & Scaiano, J. C. *Modern Molecular Photochemistry of Organic Molecules*. University Science Books, Sausalito (2010).

2. Bulović, V. *et al.* Bright, saturated, red-to-yellow organic light-emitting devices based on polarization-induced spectral shifts. *Chem. Phys. Lett.*, **287**, 455-460 (1998)

3. Parker, C.A. *Photoluminescence of Solutions*. Elsevier, Amsterdam (1968).

4. Berberan-Santos, M. N. & Garcia, J. M. M. Unusually strong delayed fluorescence of C_{70}. *J. Am. Chem. Soc.* **118,** 9391–9394 (1996).

5. Yokoyama, D. Molecular orientation in small-molecule organic light-emitting diodes, *J. Mater. Chem.* **21**, 19187-19202 (2011).

6. Komino, T. *et al.* Selectively controlled orientational order in linear-shaped thermally activated delayed fluorescent dopants. *Chem. Mater.* **26**, 3665-3671 (2014).
